# Visuomotor Training to Enhance Proprioception of Contralateral Wrist Based on the Cross‐Transfer Effect

**DOI:** 10.1111/cns.70504

**Published:** 2025-09-10

**Authors:** Yizhao Wang, Liping Huang, Zhuang Wang, Ting Liu

**Affiliations:** ^1^ Department of Rehabilitation Tianjin University Huanhu Hospital Tianjin China; ^2^ Clinical College of Neurology and Neurorehabilitation Tianjin Medical University Tianjin China; ^3^ College of Health and Exercise Science Tianjin University of Sport Tianjin China; ^4^ Academy of Medical Engineering and Translational Medicine Tianjin University Tianjin China; ^5^ College of Biomedical Engineering and Technology Tianjin Medical University Tianjin China

**Keywords:** EEG, proprioception, transfer effect, visuomotor training, wrist

## Abstract

**Background:**

Neurological diseases such as stroke or Parkinson's disease are often accompanied by weakening or loss of proprioception, which seriously affects the motor control ability of the patients. However, proprioception rehabilitation is challenging due to the pain caused by impaired joints and the hard efforts that patients have to make during training. This study investigated the cross‐transfer effect of short‐term visuomotor training to the untrained wrist from the trained wrist, from both views of behavioral results and brain activity analyses.

**Methods:**

Thirty healthy volunteers aged 25.53 ± 4.95 years were recruited for this study. They were randomly assigned into two groups: the visuomotor (*n* = 15) group performed visuomotor training (VM group) and the conventional training (*n* = 15) group (CT group) performed flexion and extension training on the right wrist. Behavioral tests (movement accuracy error, MAE) were performed both before and after training, with electroencephalogram (EEG) recorded. The movement‐related cortical potentials (MRCPs), event‐related potentials (ERPs) and event‐related spectral perturbation (ERSP) were calculated in the test session before and after training.

**Results:**

Behavioral results showed that after visuomotor training of the right wrist on the VM group, the mean MAE of both the right wrist and untrained left wrist were reduced (*p* < 0.05) after training. EEG topography showed reduced brain activity as the behavioral test task became familiar. ERPs showed a decrease in amplitude during the behavioral test for both trained and untrained wrist movements. MRCPs latency significantly increased at C3 and C4 while amplitude decreased at C3 and Cz for the right wrist; latency significantly increased at C3 while amplitude decreased at Cz for the left wrist. Increased power at CP3 for the right wrist and CP4 for the left wrist in α‐frequency ERSP after VM was observed; reduced power in β‐frequency ERSP after VM was observed for both wrists. For the CT group, the mean MAE of the trained right wrist increased (*p* < 0.05) after training, while the untrained left wrist showed no statistical significance. No significant energy changes corresponding to the left/right wrist were observed in EEG topography after training. There was a noticeable decrease in ERPs amplitude at the central and parietal regions for both the left and right wrists. The MRCPs latency significantly increased at C3 for the right wrist; however, the latency and amplitude did not significantly change for the left wrist. Decreased power at FC3 and CP3 for the right wrist in α‐frequency ERSP after CT was observed. From the regression analysis, the behavioral improvement of VM‐R was correlated with CP3; the behavioral improvement of VM‐L was correlated with C3, C4, CP3, and CP4.

**Conclusions:**

Visuomotor training on the right wrist led to proprioceptive improvements in both the trained and untrained wrists, demonstrating a cross‐transfer effect. These findings suggest that visuomotor training could be used in rehabilitation protocols to improve proprioception in patients with neurological diseases, offering a less painful and more efficient method to restore motor control.

## Graphical abstract

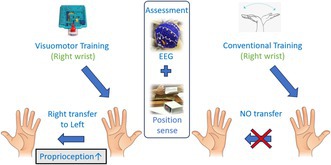

## 1 Introduction

Proprioception, the ability to sense body position and movement, is fundamental to motor control and sensorimotor integration. It is processed through a hierarchical network: mechanoreceptors transduce limb dynamics into proprioceptive inputs; in the primary somatosensory cortex (S1) and posterior parietal cortex (PPC), body schema is constructed and motor commands are refined through cerebellar‐thalamocortical loops [1, 2].

Neurological disorders such as stroke disrupt this proprioceptive hierarchy, leading to sensory deficits that impair motor function and daily activities [3]. Conventional rehabilitation strategies, such as Powerball training [4], primarily enhance spinal reflex arcs but have limited efficacy in restoring proprioceptive cortical networks [5]. In contrast, visuomotor training (VMT), which involves repetitive task‐specific practice with visual feedback, has been shown to enhance neuroplasticity in the sensorimotor cortex and may facilitate interlimb transfer of proprioceptive improvements [6].

Cross‐transfer refers to the phenomenon where training‐induced improvements in one limb transfer to the untrained contralateral limb. Proprioceptive cross‐transfer is thought to involve interhemispheric communication between the primary somatosensory cortices (S1‐S1) and PPC, facilitating the transfer of sensory information and motor commands between hemispheres [7]. PPC integrates proprioceptive inputs with efference copies to construct an internal representation of the body in space, a process essential for movement planning and execution [8, 9]. Within PPC, the superior parietal lobule (SPL) contributes to body schema formation, whereas the inferior parietal lobule (IPL) supports sensorimotor transformations required for adaptive motor control [10, 11]. In addition to its role in body schema construction, PPC is reciprocally connected with frontal areas, particularly the dorsal premotor cortex (PMd), forming circuits that transform sensory inputs into goal‐directed motor actions [12, 13]. These interactions are critical for sensorimotor learning and movement adaptation [14, 15]. PMd is involved in movement preparation, decision making, and motor planning, which are essential for proprioceptive learning [16, 17]. Furthermore, proprioceptive cross‐transfer may involve interhemispheric interactions between S1 and M1, reflecting the plasticity of somatosensory and motor networks [6, 18]. These neural mechanisms highlight the importance of M1, S1, PPC, and PMd in proprioceptive cross‐transfer, providing a foundation for understanding how training‐induced plasticity in one limb can influence the contralateral limb.

Electroencephalography (EEG) provides a valuable tool for investigating the cortical mechanisms underlying proprioceptive cross‐transfer. Specifically, EEG can measure cortical oscillatory changes associated with sensory adaptation and interhemispheric communication. This includes movement‐related cortical potentials (MRCPs), which provide insight into the preparatory motor activity [19]; event‐related potentials (ERPs), which examine sensory and cognitive processing, particularly in response to proprioceptive stimuli [20]; and event‐related spectral perturbation (ERSP), which quantifies changes in cortical oscillatory activity [21]. Our study combines behavioral assessments of proprioceptive acuity with multi‐dimensional measures (MRCPs, ERPs, and ERSP) to investigate the cortical mechanisms underlying proprioceptive cross‐transfer induced by VMT. We hypothesize that unilateral VMT will enhance proprioceptive acuity in both the trained and untrained limbs, mediated by training‐induced changes in cortical oscillatory activity reflecting sensory adaptation and interhemispheric communication.

## 2 Methods

### 2.1 Subjects

While the ultimate goal of this research is to inform rehabilitation strategies for neurological patients, the present study focuses on healthy participants to establish a foundational understanding of proprioceptive cross‐transfer mechanisms in an intact sensorimotor system. By eliminating confounding factors such as lesion location or severity, this approach allows us to isolate the effects of VMT on proprioceptive adaptation. These findings may contribute to the development of more effective proprioceptive training strategies, particularly for clinical populations with neurological deficits.

Thirty healthy volunteers (25.53 ± 4.95 years of age, ranging from 18 to 35 years) were recruited and randomly assigned to two groups: (A) the cross‐transfer group (*n* = 15) performed visuomotor training (VM group) on the right wrist; (B) the conventional training (*n* = 15) group (CT group) performed flexion and extension training on the right wrist. G*Power was used for sample size estimation. The parameters used for the calculation refer to the appendix. All participants were right‐handed according to the Edinburgh Handedness Inventory and had no history of neurological diseases or forearm injuries. All participants signed informed consent before participating in the study. The study was approved by the relevant ethics committees of Tianjin University of Sport, China, and Tianjin Huanhu Hospital, China.

### 2.2 Apparatus

#### 2.2.1 Custom‐Made Wrist Proprioception Evaluation Device

**FIGURE 1:**
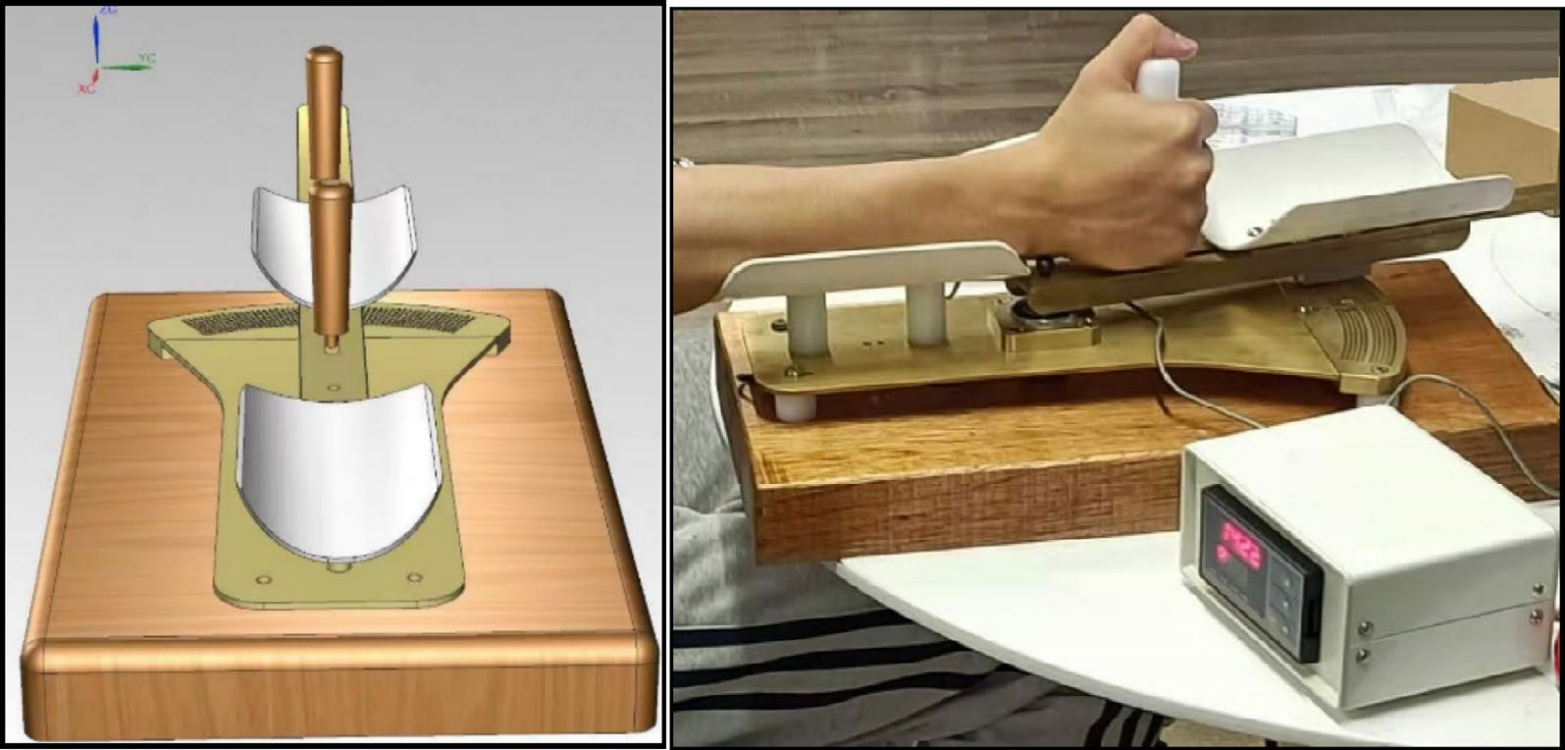
Custom‐made wrist proprioception evaluation device.

The custom‐made wrist evaluation device (Figure 1) is a one‐degree‐of‐freedom manipulandum which was used to assess and quantify proprioceptive and motor function before and after visuomotor/conventional training. During the experiment, the participants grasped the handle, with the starting position being their hand and forearm positioned straight forward. The horizontal angle was displayed on an external monitor with an accuracy of 0.01°. The validity and reliability of this proprioceptive evaluation device have been verified. The Intraclass Correlation Coefficient (ICC) data, demonstrating the device's reliability, are reported in the Supporting Information S1. For further details on the reliability and validity study of this device, please refer to the appendix.

#### 2.2.2 Wrist‐Manipulated 3D Maze

**FIGURE 2:**
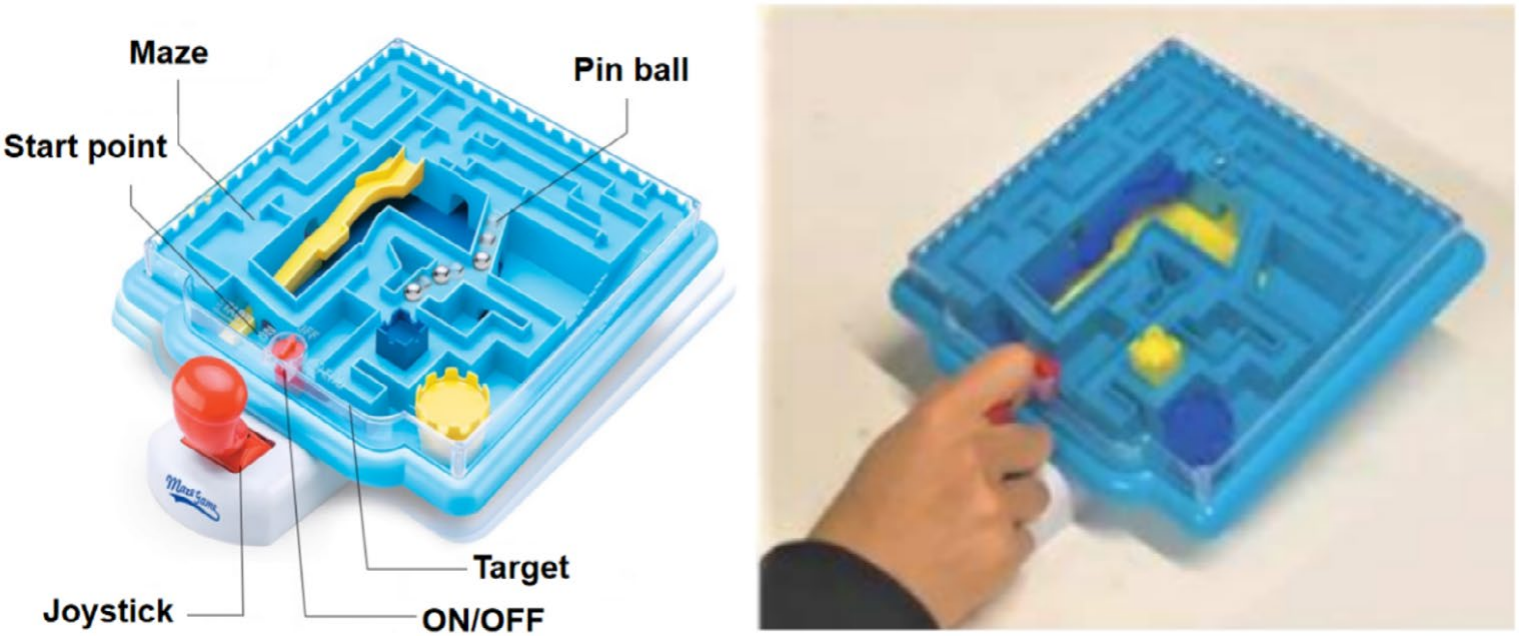
Wrist‐manipulated 3D maze. A wrist‐operated joystick can tilt the panel at different angles, allowing the participant to guide the pin ball through the maze.

The wrist‐manipulated 3D maze (MazeGames, Zhejiang, China) was used for visuomotor training (Figure 2). The 3D maze is controlled through flexion, extension, ulnar deviation, and radial deviation of the wrist. These wrist movements manipulate a control stick, which in turn tilts the panel left, right, forward, or backward. Owing to the effect of gravity, tilting the panel causes a small ball inside the maze to move. The training objective is to guide the ball from the starting point to the end point of the maze with repeated movements.

### 2.3 Experimental Sessions and EEG Recording

**FIGURE 3:**
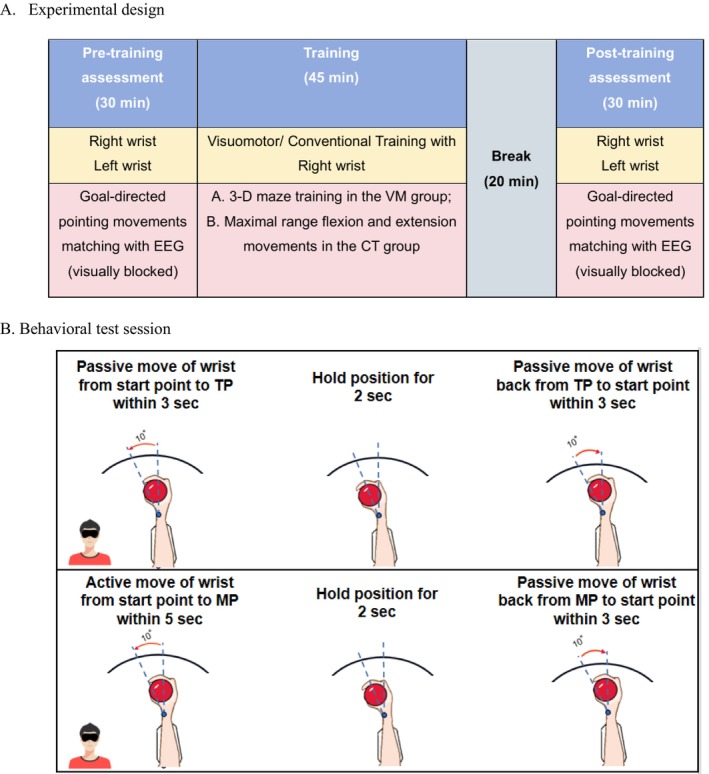
Schemata of the experimental design (A) and the procedure of the behavioral test sessions (B). The participants were visually blocked during the behavioral tests.

The experiment consisted of two assessments and one training session, all completed on the same day (Figure 3A). First, the right and left wrists were evaluated by behavioral assessment and EEG monitoring (Figure 3B). Then, the participants performed visuomotor/conventional training on the right wrist for 45 min. At last, the behavioral assessment and EEG monitoring were repeated after 20 min rest.

#### 2.3.1 Training Session

Two breaks of 2 min each were allowed during the training to prevent fatigue. Both training modalities were conducted with eyes open and without visual occlusion.

##### 2.3.1.1 Visuomotor Training

The participants engaged in visuomotor training via the 3D maze, which entails quick, precise, small‐amplitude movements of the right wrist under visual guidance, including flexion, extension, adduction, and abduction movements.

##### 2.3.1.2 Conventional Flexion and Extension Training

In the resting state, the participants' right forearm was naturally relaxed and placed flat on the table. During training, the participant performs maximal range flexion and extension movements of the wrist without any assistance, repeating this motion at a frequency of 10 repetitions per minute.

#### 2.3.2 Pre/Post‐Training Behavioral Test Session (Behavioral Assessment)

##### 2.3.2.1 Passive Move to the Target Position (TP)

The wrist flexion TP was set to 10°. The study has confirmed that joint position sense is one of the key components of proprioception that decreases in accuracy as the joint angle increases [22]. In previous studies, the target position usually chosen was 15° [23, 24]. Therefore, in this study, a smaller angle of 10° was selected, and this angle has been tested in a preliminary experiment. The right wrist was moved by the experimenter at a constant speed (6°/s) [23, 24] to reach the target within 3 s, according to the screen prompt, and then held there for 2 s. During this time, another experimenter recorded data via an external monitor. The wrist was then brought back to the neutral position at a constant speed, taking 3 s. There was a 6‐s break between trials.

##### 2.3.2.2 Active Move to the Matching Position (MP)

The behavioral test was performed according to the matching task paradigm. Resent research indicates that an increasing number of studies have been employing joint position matching as a useful tool with acceptable validity and reliability [25]. This is a well‐established paradigm [24]. In this experiment, sounds were used to prompt the patients to perform active wrist movements. When the stimulus paradigm finishes playing the instruction “Please perform an active movement”, the subjects start to carry out the active movement, and the stimulus interface marks a trigger point at that moment. The participants perceived the TP through proprioception and then actively flexed the wrist to match the perceived TP within 5 s, repeatedly adjusting the wrist flexion angle until they believed they reached the TP and holding it for 2 s. The rotation angle was recorded. The wrist was then brought back to the starting position within 3 s by the experimenter. Ten sets of matching tasks were performed by each participant. The average absolute value of the differences between the TP and the MP was calculated as the movement accuracy error (MAE), which represents the sensorimotor function of the wrist. MAE indicates systematic error (i.e., bias), which was calculated as the mean difference between the matching and reference position. Position sense error variability (i.e., SD_error_) indicates random error (precision), which was computed as the standard deviation of the MAE [26].

Both passive and active movements were performed for both the trained wrist (right wrist) and the contralateral wrist (left wrist) to evaluate position sense with EEGs recorded. The matching procedure was repeated for 10 trials. The participants wore opaque eye patches to block visual inputs during the behavioral tests. All participants underwent a familiarization phase before baseline testing.

#### 2.3.3 EEGs Recording and Preprocessing (Mechanism Assessment)

**FIGURE 4:**
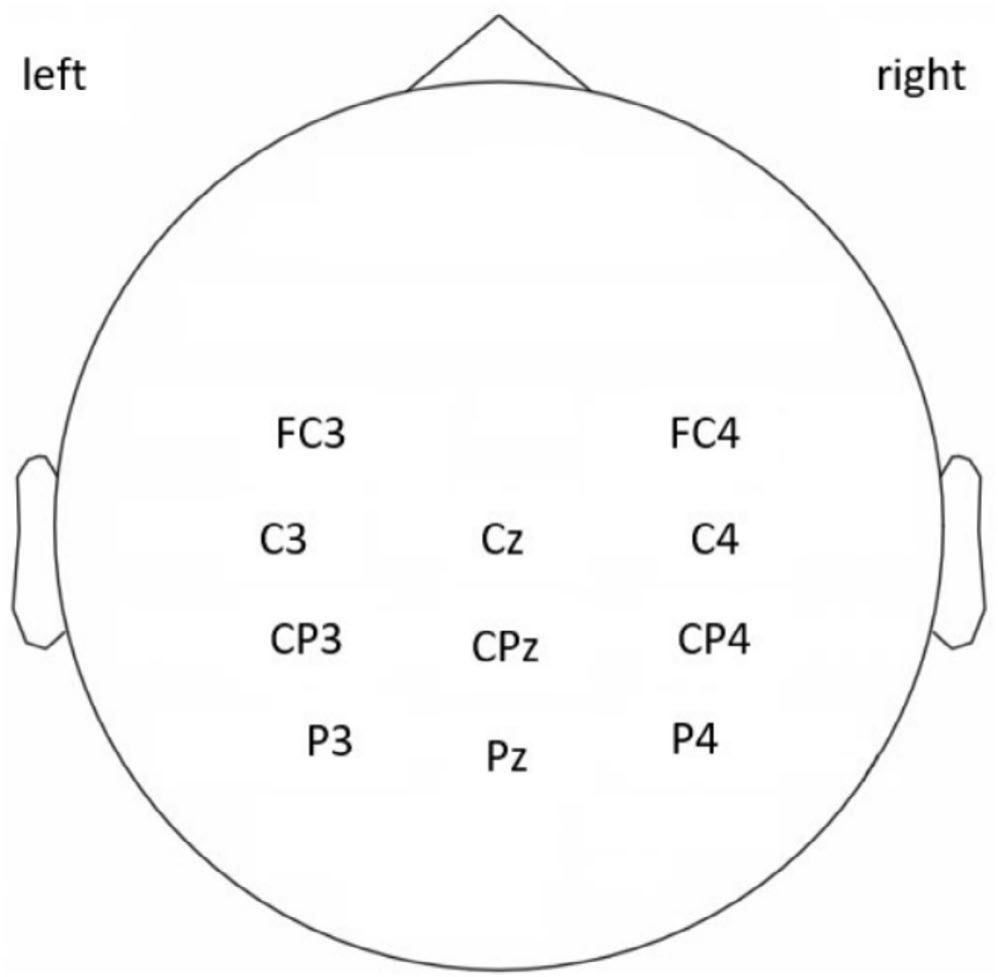
Placement of electrodes. EEGs were recorded at FC3, FC4, C3, Cz, C4, CP3, CPz, CP4, P3, Pz, and P4.

EEGs were recorded with Neuroscan (El Paso, TX, USA) via a 32‐channel Quick‐cap (Ag/AgCl electrodes) according to the international 10/20 electrode system with a sampling rate of 1024 Hz. As this study focused on M1, S1, PPC, and PMd (Table 1), which correspond to the proprioceptive cross‐transfer associated with the wrists, 11 electrodes were selected (labeling FC3, FC4, C3, Cz, C4, CP3, CPz, CP4, P3, Pz, P4 as shown in Figure 4) for analysis. The reference electrode was placed in the middle of Cz and CPz, and the ground electrode was placed on the forehead. All the signals were filtered (0.5–100 Hz) and notch‐filtered (49–51 Hz). The data was re‐referenced to the common median reference, followed by removal of transient high‐amplitude artifacts with independent component analysis (ICA). Then the denoised EEGs were epoched (−500 to 1500 ms) into trials, with 0 ms aligned to the onset of the sound cue at the beginning of each trial [27]. A baseline correction was applied using the −500 to 0 ms pre‐sound cue onset.

**TABLE 1:** The corresponding relationship between cortical areas and EEG electrodes.

Cortical areas	Electrodes
Dorsal premotor cortex (PMd)	FC3, FC4
Primary motor cortex (M1) & primary somatosensory cortex (S1)	C3, C4
Sensorimotor integration zone (midline M1/S1)	Cz
S1 & PPC (sensorimotor cortex)	CP3, CPz, CP4
Posterior parietal cortex (PPC)	P3, Pz, P4

The EEG analysis and comparison in this study was aimed at the process of active matching position.

##### 2.3.3.1 EEG Topographical Distribution Maps

The phenomenon of event‐related desynchronization (ERD) was observed. EEG topographical distribution maps were drawn to observe the effects of visuomotor/conventional training on the training side and on the opposite side of the brain regions as a whole.

##### 2.3.3.2 Event‐Related Potentials (ERPs) and Movement‐Related Cortical Potentials (MRCPs)

ERPs and MRCPs [28, 29] were computed during behavioral tests before and after training within a time window from 500 ms prior to and 1500 ms after stimulus onset for both groups. The 500 ms pre‐test interval served as the baseline. ERPs within the 0 to 1000 ms time window were analyzed across the central and parietal regions (C3, Cz, C4, CP3, CPz, CP4, P3, Pz, and P4). The latency and amplitudes of MRCPs were analyzed from 0 to 1000 ms in central areas (C3, Cz, and C4). MRCPs represent movement intention, preparation, and execution, indicating top‐down modulation of proprioceptive processing [30]. Since MRCP was highly correlated with movement, the electrodes C3 and C4 were selected for the study. ERPs reveal higher‐order integration in the PPC, capturing global network interactions [31]. Therefore, in the current study, the CP3, CP4, P3, and P4 electrodes were selected as the primary objects of observation.

##### 2.3.3.3 Event‐Related Spectral Perturbation (ERSP)

ERSP analysis is a time‐frequency transform that computes the change in signal power at each frequency from baseline for an event to represent event‐related changes in activity. ERS (Event‐Related Synchronization) and ERD (Event‐Related Desynchronization) were observed. ERSP reflects local cortical excitability changes [32]. Since this study mainly involves sensation and movement, the electrodes FC3, FC4, C3, C4, CP3, and CP4 were selected. α‐band (8–13 Hz), which is known to play a role in attentional modulation and sensorimotor integration [33]. β‐frequency (14–25 Hz) has long been recognized as sensorimotor rhythms indicative of movement preparation and planning [32, 34]. The ERSP was computed as average event‐related variation across trials (in dB) compared to the respective baseline (500 ms prior to the sound cue) [35, 36]. The equation is as follows [37]:

(1) $$\text{ERSP}\;\left(f,t\right)\;=\;\frac{1}{n}\;\sum_{k=1}^{n}\left(F_{k}\;{\left(f,t\right)}^{2}\right)$$

where, $F_{k}\;\left(f,t\right)$ defines the spectral estimation of the kth trial at frequency *f* within frequency and time *t*, which was computed using wavelet transform with Morlet wavelets.

### 2.4 Statistical Analysis

Validity and reliability tests for the custom‐made wrist proprioception evaluation device were conducted with the intraclass correlation coefficient (ICC) and Pearson correlation coefficient, respectively. Outliers were defined as values above or below 1.5 times the interquartile range (IQR) [38]. All the data were examined for normality via the Shapiro–Wilk test. Nonnormally distributed data were logarithmically transformed and then retested for normality. Paired *t*‐test were performed for both wrists at post‐training relative to pre‐training. Perform a regression analysis using the MAE as the dependent variable and the 9 electrodes in the ERP analysis as the independent variables. G*Power (3.1.9.2) was used for sample size estimation. All the statistical analyses were conducted via SPSS (version 24.0) and R studio (version 1.1.456). The data in the text and figures are expressed as the means ± SD. The *p* value was considered statistically significant at *p < 0.05.

## 3 Results

### 3.1 VM Group

#### 3.1.1 Behavioral Evidence of Sensory‐Motor Learning of the Right/Left Wrist After VM Training

**FIGURE 5:**
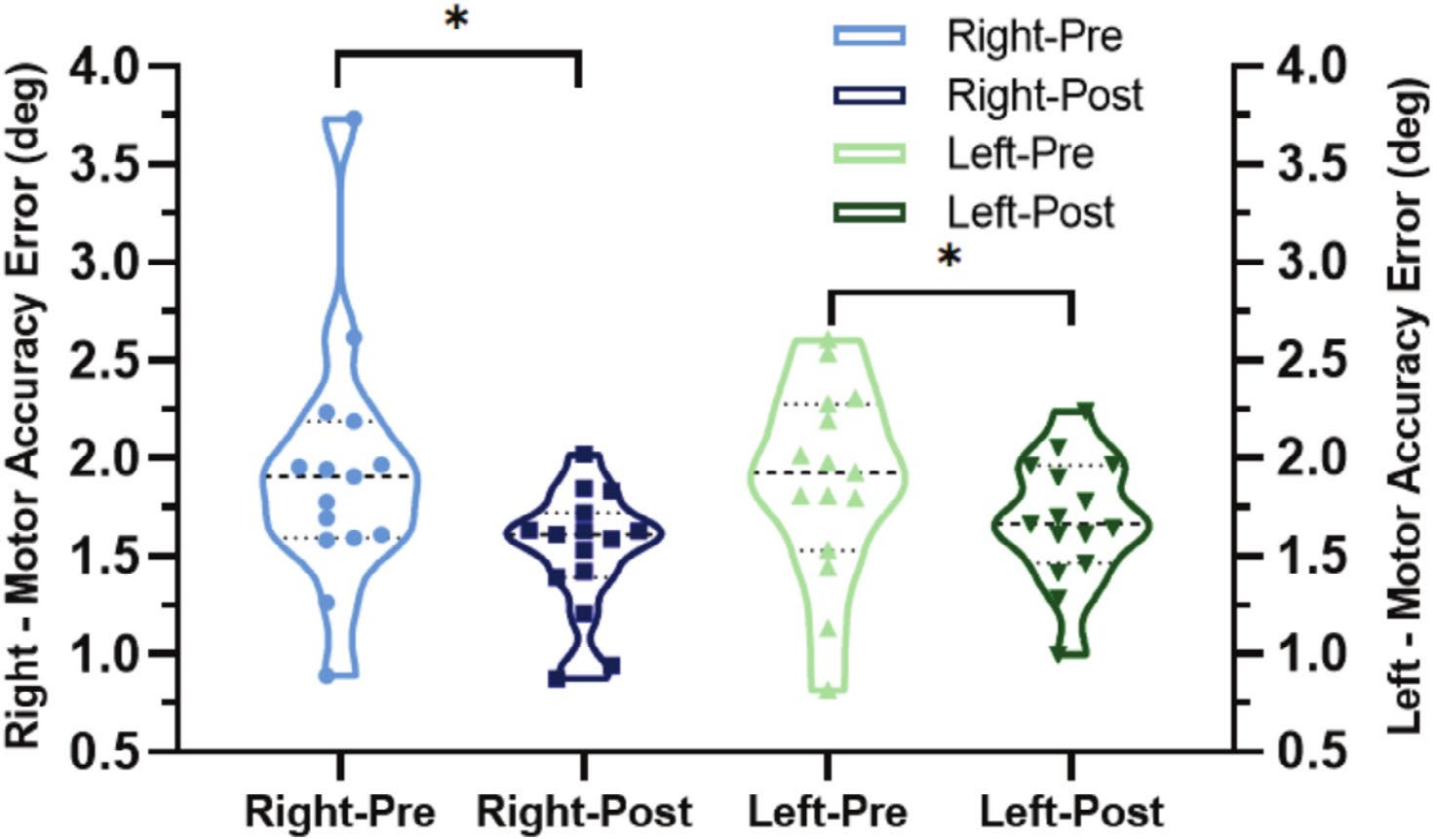
Violin plot indicating the distribution of the MAE at pre‐/post‐training for the right/left wrist of the VM group. Dot lines represent the 25th and 75th percentiles. Dash line represents the median. Each point in the violin plot represents one participant (*p < 0.05).

The movement accuracy error (MAE) of all participants was compared between pre‐training and post‐training to evaluate the effects of the VM training, which involved manipulating the 3D maze, on the trained right wrist and both ipsilateral and contralateral wrists. The mean MAE of the right wrist decreased from 1.93° ± 0.65° to 1.52° ± 0.32° after training. Similarly, the mean MAE of the left wrist decreased from 1.88° ± 0.50° to 1.69° ± 0.32° after training. Both wrists exhibited a significant mean relative change, with the trained right wrist showing a 21% decrease (*t* = 2.727, *p* = 0.016) and the contralateral left wrist demonstrating a 10% decrease (*t* = 2.246, *p* = 0.041, Table 2, Figure 5). The mean variation (SD_error_) of the left wrist decreased from 1.64° ± 0.42° to 1.22° ± 0.35° after training, which was a 25% decrease (*t* = 2.588, *p* = 0.021).

**TABLE 2:** MAE (deg) and SD_error_ of the right/left wrist for VM group.

	Pre	Post	*p*	Effect size	Power
Right‐MAE	1.93 ± 0.65	1.52 ± 0.32	0.016*	0.725	0.970
Right‐SD_error_	1.20 ± 0.32	0.95 ± 0.25	0.083	0.839	0.993
Left‐MAE	1.88 ± 0.50	1.69 ± 0.32	0.041*	0.431	0.625
Left‐SD_error_	1.64 ± 0.42	1.22 ± 0.35	0.021*	1.086	1.000

* *p* < 0.05.

These results indicate that short‐term VM training of the right wrist significantly enhanced movement accuracy for both the trained and untrained wrists, suggesting that cross‐transfer of proprioceptive learning occurs from the trained wrist to the contralateral wrist.

#### 3.1.2 EEG Topographical Maps Evidence of Sensory‐Motor Learning of the Right/Left Wrist After VM Training

**FIGURE 6:**
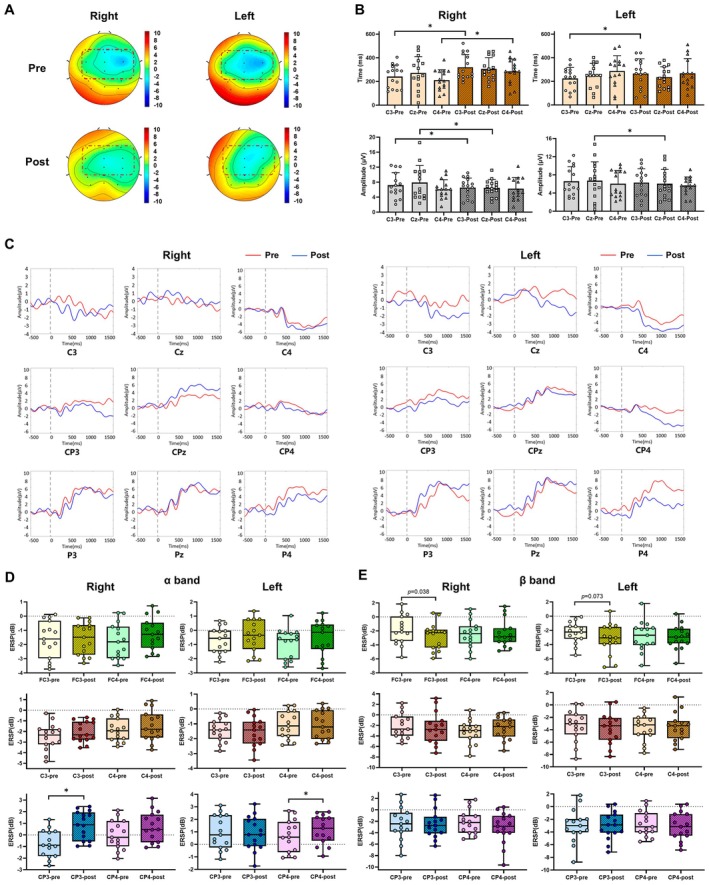
(A) Topographical 2D scalp maps at pre‐/post‐training for the left/right wrist of the VM group. Red box: sensorimotor area of the parietal region. (B) MRCPs latency and amplitude at C3, Cz, and C4 pre‐/post‐training for the right/left wrist of the VM group. Each point in the bar chart represents one participant (*p < 0.05). (C) ERPs (C3, Cz, C4, CP3, CPz, CP4, P3, Pz, and P4) at pre‐/post‐training for the right/left wrist of the VM group. Red line: pre‐training. Blue line: post‐training. Dash line: trigger. (D) α‐frequency ERSP (FC3, FC4, C3, C4, CP3, and CP4) at pre‐/post‐training for the right/left wrist of the VM group. Each point in the box plot represents one participant (*p < 0.05). (E) β‐frequency ERSP (FC3, FC4, C3, C4, CP3, and CP4) at pre‐/post‐training for the right/left wrist of the VM group. Each point in the bar chart represents one participant (*p < 0.05).

Prior to the VM training, active movements of both the left and right wrists resulted in heightened EEG activity within the sensorimotor region of the parietal cortex. Following training, as participants became more familiar and proficient with the tasks, the required effort decreased, resulting in diminished EEG activity (Figure 6A). The results for both the right and left wrists aligned with the behavioral indicators (Table 2), demonstrating an improvement in movement accuracy.

#### 3.1.3 MRCPs Evidence of Sensory‐Motor Learning of the Right/Left Wrist After VM Training

As illustrated in Figure 6B, the latency of MRCPs significantly increased at C3 and C4 (*p* < 0.05), whereas the amplitude decreased at C3 and Cz (*p* < 0.05) for the right wrist. Additionally, latency significantly increased at C3, and amplitude decreased at Cz (*p* < 0.05) for the left wrist (Table 3).

**TABLE 3:** MRCPs changes for the VM group.

	C3		Cz		C4	
	Latency (ms)	Amplitude (μV)	Latency (ms)	Amplitude (μV)	Latency (ms)	Amplitude (μV)
Pre‐training						
VM‐R	242.23 ± 97.22	7.18 ± 3.38	271.83 ± 138.89	7.92 ± 4.54	212.18 ± 87.04	6.13 ± 2.52
VM‐L	224.88 ± 92.36	6.61 ± 3.21	251.30 ± 100.97	6.73 ± 4.16	287.60 ± 129.50	6.07 ± 2.93
Post‐training						
VM‐R	320.75 ± 107.39*	6.56 ± 2.55*	305.78 ± 95.27	6.51 ± 2.20*	286.15 ± 103.82*	6.27 ± 2.95
VM‐L	266.70 ± 124.44*	6.31 ± 3.05	235.95 ± 88.32	6.06 ± 3.14*	268.90 ± 124.26	5.66 ± 1.89

*Note:* Mean ± SD.

* *p* < 0.05.

#### 3.1.4 ERPs Evidence of Sensory‐Motor Learning of the Right/Left Wrist After VM Training

The post‐training analysis of the VM group revealed changes in the amplitude of event‐related potentials (ERPs) for both the right and left wrists within the 0–1000 ms time window. For the right wrist, a noticeable decrease in ERP amplitude was observed in the central and parietal regions (C3, CP3, P3, and P4; Figure 6C). Similarly, a noticeable decrease in ERP amplitude was noted in the central and parietal regions (C3, CP3, C4, and P4; Figure 6C) of the left wrist.

#### 3.1.5 α‐Frequency ERSP Evidence of Sensory‐Motor Learning of the Right/Left Wrist After VM Training

Increased power at CP3 for the right wrist and CP4 for the left wrist in α‐frequency ERSP after VM was observed (Figure 6D).

#### 3.1.6 β‐Frequency ERSP Evidence of Sensory‐Motor Learning of the Right/Left Wrist After VM Training

Reduced power at FC3 in β‐frequency ERSP after VM was observed for both wrists (Figure 6E).

### 3.2 CT Group

#### 3.2.1 Behavioral Evidence of Sensory‐Motor Learning of the Right/Left Wrist After CT

**FIGURE 7:**
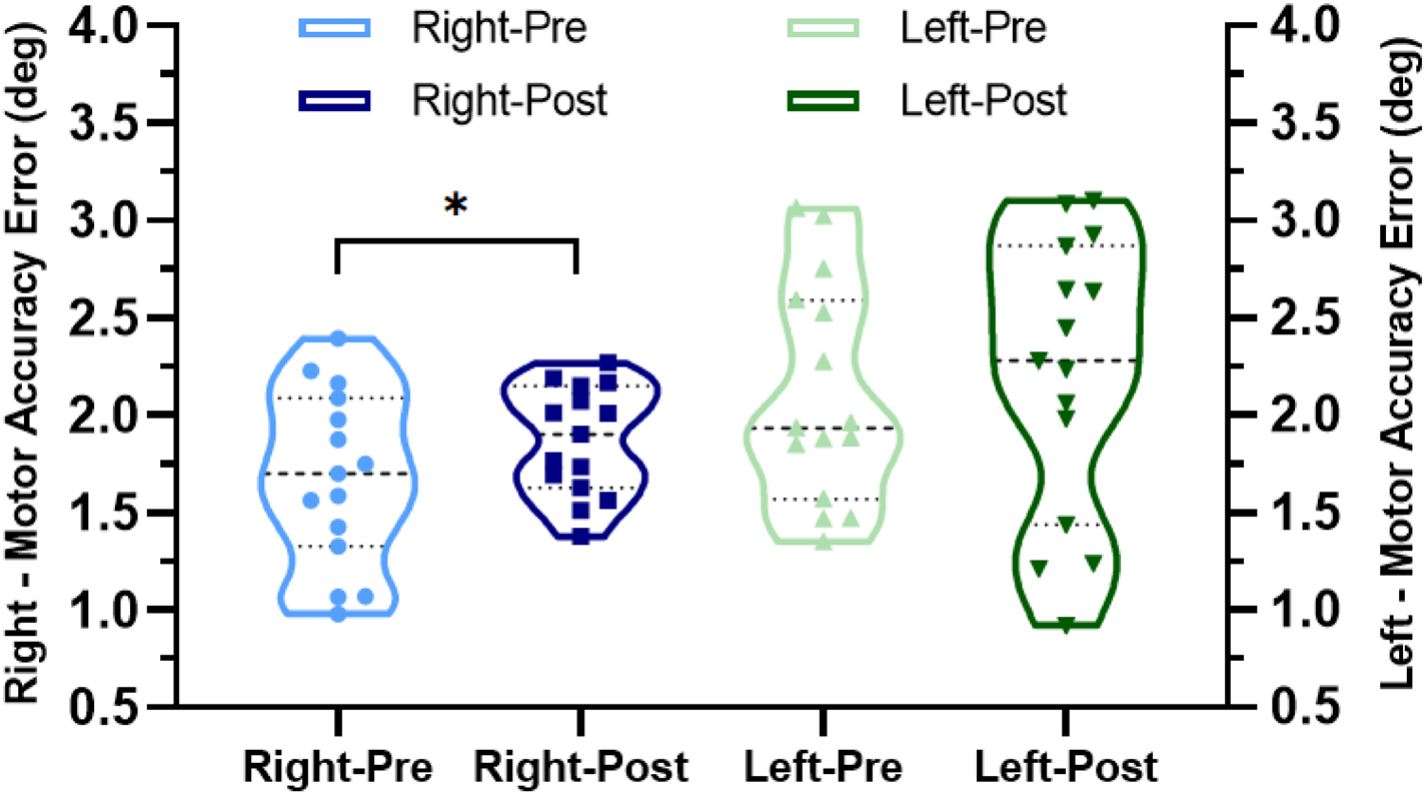
Violin plot indicating the distribution of the MAE at pre‐/post‐training for the right/left wrist of the CT group. Dot lines represent the 25th and 75th percentiles. Dash line represents the median. Each point in the violin plot represents one participant (*p < 0.05).

The mean MAE of the right wrist increased from 1.68° ± 0.45° to 1.87° ± 0.28°, indicating a statistically significant decrease in motor function (trained right wrist showing an 11% increase, *t* = −2.343, *p* = 0.034). The mean MAE of the left wrist and SD_error_ for both wrists were not significantly different after training (Table 4, Figure 7).

**TABLE 4:** MAE (deg) and SD_error_ of the right/left wrist for the CT group.

	Pre	Post	*p*	Effect size	Power
Right‐MAE	1.68 ± 0.45	1.87 ± 0.28	0.034*	0.486	0.730
Right‐SD_error_	1.37 ± 0.51	1.19 ± 0.20	0.234	0.403	0.570
Left‐MAE	2.11 ± 0.57	2.21 ± 0.72	0.589	0.154	0.129
Left‐SD_error_	1.39 ± 0.39	1.32 ± 0.41	0.542	0.168	0.144

* *p* < 0.05.

#### 3.2.2 EEG Topographical Maps Evidence of Sensory‐Motor Learning of the Right/Left Wrist After CT

**FIGURE 8:**
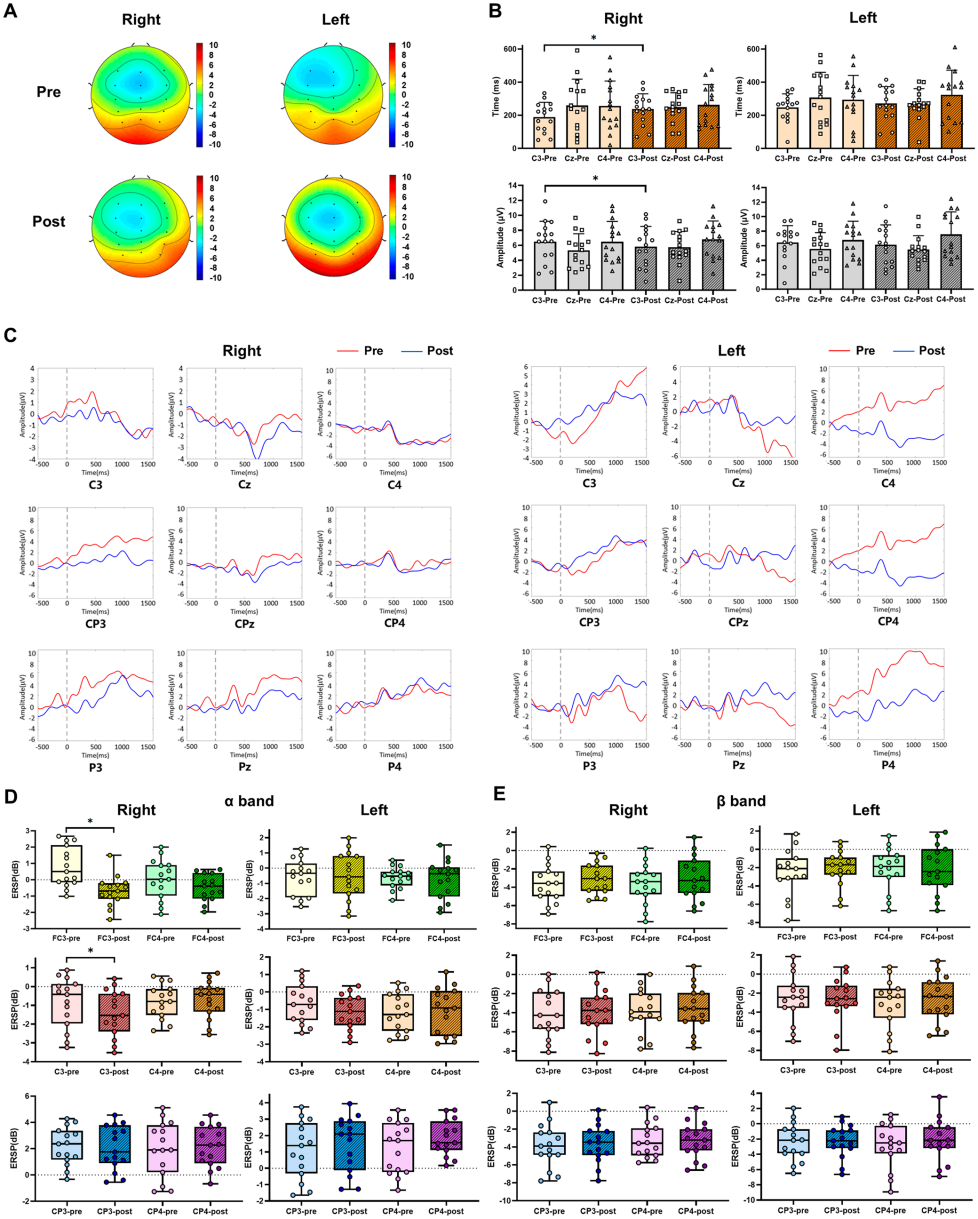
(A) Topographical 2D scalp maps at pre‐/post‐training for the left/right wrist of the CT group. (B) MRCPs latency and amplitude at C3, Cz, and C4 pre‐/post‐training for the right/left wrist of the CT group. Each point in the bar chart represents one participant (*p < 0.05). (C) ERPs (C3, Cz, C4, CP3, CPz, CP4, P3, Pz, and P4) at pre‐/post‐training for the right/left wrist of the CT group. Red line: Pre‐training. Blue line: Post‐training. Dash line: Trigger. (D) α‐frequency ERSP (FC3, FC4, C3, C4, CP3, and CP4) at pre‐/post‐training for the right/left wrist of the CT group. Each point in the bar chart represents one participant (*p < 0.05). (E) β‐frequency ERSP (FC3, FC4, C3, C4, CP3, and CP4) at pre‐/post‐training for the right/left wrist of the CT group. Each point in the bar chart represents one participant (*p < 0.05).

No significant energy changes corresponding to the left or right wrists were observed in the EEG topography when pre‐training and post‐training measurements were compared (Figure 8A).

#### 3.2.3 MRCPs Evidence of Sensory‐Motor Learning of the Right/Left Wrist After CT

As illustrated in Figure 8B, the latency of MRCPs significantly increased at C3 (*p* < 0.05), whereas the amplitude decreased at the same location for the right wrist; however, latency and amplitude showed no statistically significant changes for the left wrist (Table 5).

**TABLE 5:** MRCPs changes for the CT group.

	C3		Cz		C4	
	Latency (ms)	Amplitude (μV)	Latency (ms)	Amplitude (μV)	Latency (ms)	Amplitude (μV)
Pre‐training						
CT‐R	189.50 ± 88.95	6.51 ± 2.70	259.17 ± 159.05	5.34 ± 2.21	256.40 ± 150.72	6.48 ± 2.71
CT‐L	248.10 ± 81.97	6.46 ± 2.30	307.90 ± 151.58	5.56 ± 2.24	294.33 ± 146.79	6.79 ± 2.58
Post‐training						
CT‐R	237.20 ± 92.73*	5.84 ± 2.69*	248.57 ± 87.47	5.75 ± 2.08	262.53 ± 123.10	6.80 ± 2.44
CT‐L	272.60 ± 100.77	6.15 ± 2.72	271.80 ± 88.89	5.49 ± 1.89	323.97 ± 148.28	7.57 ± 3.09

*Note:* Mean ± SD.

* *p* < 0.05.

#### 3.2.4 ERPs Evidence of Sensory‐Motor Learning of the Right/Left Wrist After CT

Post‐training analysis of the CT group revealed changes in the amplitude of ERPs for both the right and left wrists within a time window of 0 to 1000 ms. For the right wrist, a noticeable decrease in ERP amplitude was observed in the central and parietal regions (C3, CP3, P3, and Pz, Figure 8C). Similarly, a noticeable decrease in ERP amplitude was noted in the central and parietal regions (C4, CP4, and P4, Figure 8C).

#### 3.2.5 α‐Frequency ERSP Evidence of Sensory‐Motor Learning of the Right/Left Wrist After CT

Decreased power at FC3 and CP3 for the right wrist in α‐frequency ERSP after CT was observed (Figure 8D).

#### 3.2.6 β‐Frequency ERSP Evidence of Sensory‐Motor Learning of the Right/Left Wrist After CT

Although no statistically significant changes in power were observed in the β‐frequency ERSP following CT for either wrist, an increasing trend in neural activity was noted at FC3 for both wrists (Figure 8F).

### 3.3 Intra−/Inter‐Group Comparisons of Latency and Amplitude for the VM and CT Group

#### 3.3.1 Intra−/Inter‐Group Comparisons of Latency for the VM and CT Group

**FIGURE 9:**
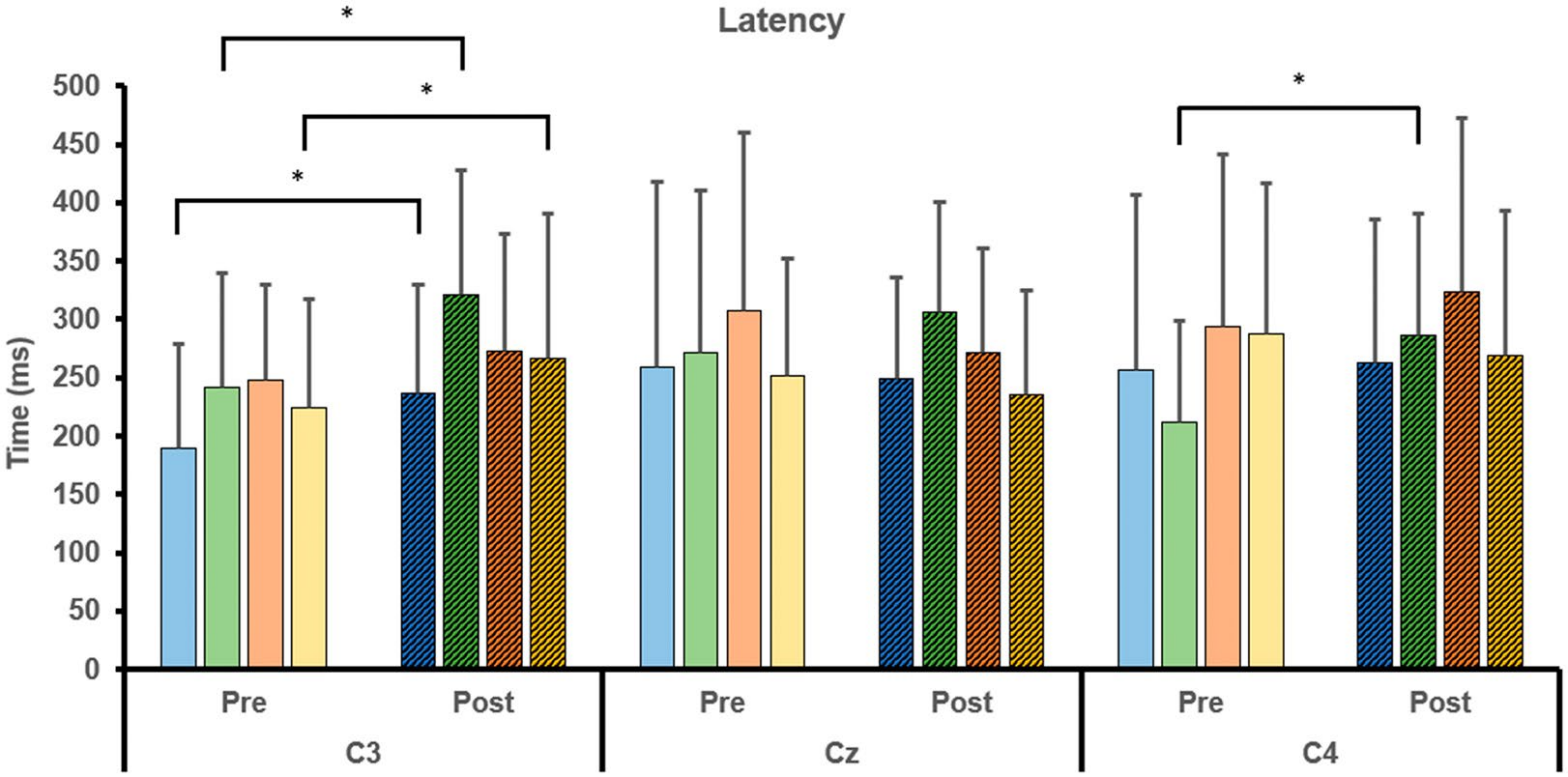
Intra‐/inter‐group comparisons of latency for the VM and CT group. 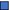 CT, right wrist; 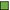 VM, right wrist; 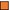 CT, left wrist; and 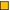 VM, left wrist (*p < 0.05).

In the intra‐group comparison (Figure 9), the latency significantly increased after training at C3 for the right wrist in the conventional training (CT) group (*p* < 0.05), at C3 for both wrists in the visuomotor (VM) group (*p* < 0.05), and at C4 for the right wrist in the VM group (*p* < 0.05). No statistically significant changes were observed in the inter‐group comparison.

#### 3.3.2 Intra−/Inter‐Group Comparisons of Amplitude for the VM and CT Group

**FIGURE 10:**
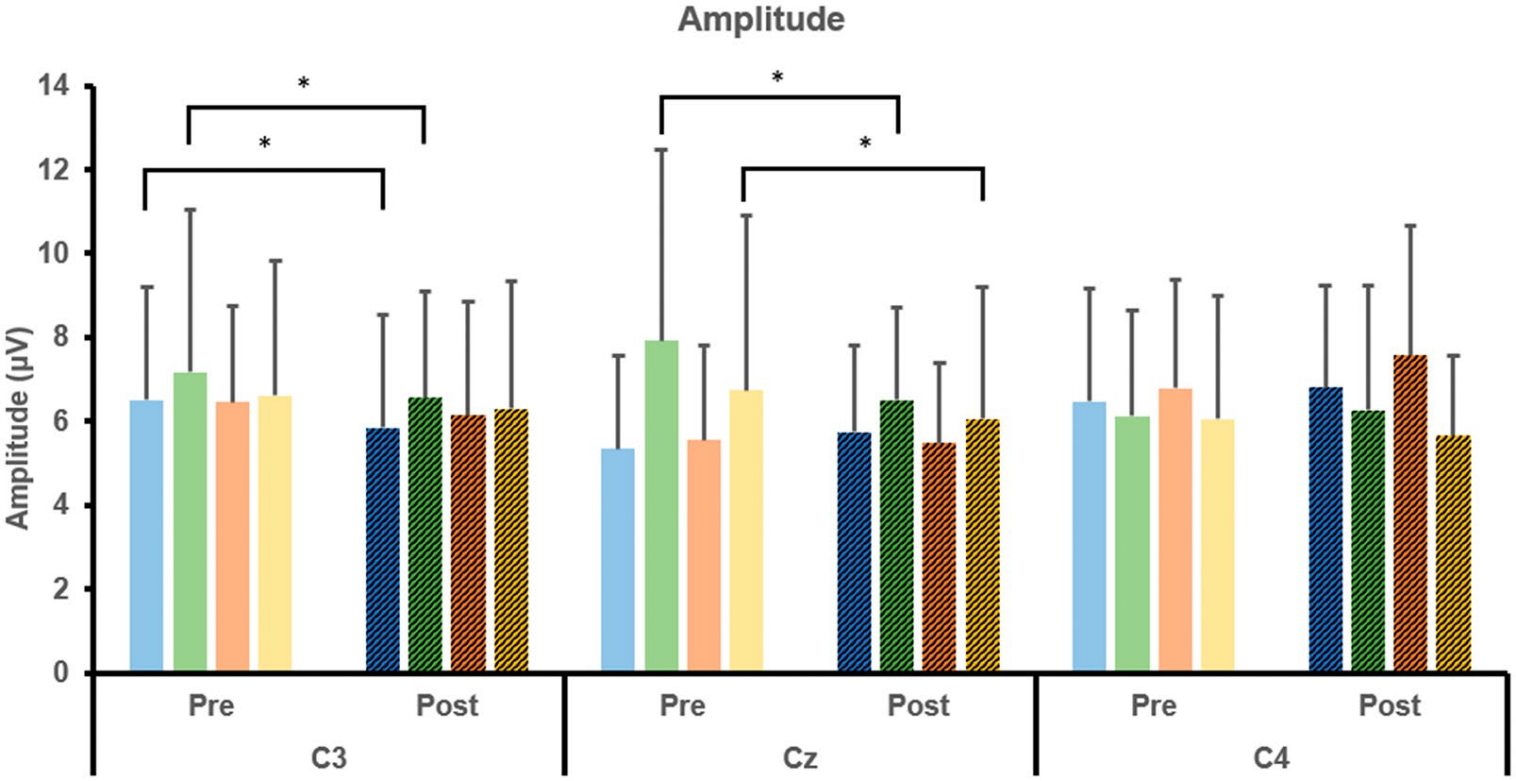
Intra‐/inter‐group comparisons of amplitude for the VM and CT group. 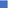 CT, right wrist; 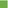 VM, right wrist; 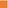 CT, left wrist; and 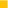 VM, left wrist (*p < 0.05).

In the intra‐group comparison (Figure 10), the amplitude significantly decreased after training at C3 for the right wrist in both the CT and VM groups (*p* < 0.05), and at Cz for both wrists in the VM group (*p* < 0.05). No statistically significant changes were observed in the inter‐group comparison.

### 3.4 Relationship Between ERP and Behavioral Changes

Behavioral results showed that for VM‐R and VM‐L, the MAE significantly decreased. In the CT‐R group, the MAE significantly increased. Therefore, using MAE as the dependent variable and the 9 electrodes in the ERP analysis as the independent variables, three regression analyses were conducted. The results showed that the behavioral improvement of VM‐R was correlated with CP3; the behavioral improvement of VM‐L was correlated with C3, C4, CP3, and CP4; and the behavioral deterioration of CT‐R was not correlated with the selected electrodes (Table 6).

**TABLE 6:** Regression analyses between ERP changes and MAE.

Model		Unstandardized coefficients		Standardized coefficients	*t*	*p*	95.0% confidence interval for B	
		B	Standard error	Beta			Lower bound	Upper bound
VM‐R	(Constant)	−0.407	0.638		−0.639	0.551	−2.047	1.232
	C3	0.119	0.158	0.176	0.752	0.486	−0.288	0.526
	Cz	0.526	0.426	0.410	1.234	0.272	−0.570	1.621
	C4	0.073	0.306	0.050	0.238	0.822	−0.714	0.859
	CP3	1.019	0.260	0.915	3.916	0.011*	0.350	1.688
	CPz	0.405	0.197	0.608	2.056	0.095	−0.101	0.911
	CP4	0.236	0.233	0.185	1.010	0.359	−0.364	0.835
	P3	−0.480	0.251	−0.487	−1.908	0.115	−1.126	0.166
	Pz	0.199	0.248	0.155	0.801	0.460	−0.439	0.836
	P4	0.002	0.067	0.004	0.027	0.980	−0.170	0.173
VM‐L	(Constant)	0.513	0.084		6.072	0.002	0.296	0.730
	C3	0.201	0.037	0.500	5.361	0.003*	0.105	0.297
	Cz	−0.066	0.065	−0.092	−1.020	0.354	−0.232	0.100
	C4	0.188	0.023	0.725	8.249	0.000*	0.129	0.246
	CP3	0.077	0.018	0.299	4.222	0.008*	0.030	0.124
	CPz	−0.171	0.110	−0.166	−1.562	0.179	−0.453	0.110
	CP4	0.069	0.026	0.230	2.662	0.045*	0.002	0.136
	P3	0.039	0.033	0.106	1.189	0.288	−0.045	0.123
	Pz	0.032	0.041	0.074	0.773	0.474	−0.074	0.138
	P4	0.037	0.024	0.167	1.566	0.178	−0.024	0.097
CT‐R	(Constant)	0.474	3.565		0.133	0.899	−8.690	9.638
	C3	−0.686	1.026	−0.443	−0.668	0.533	−3.324	1.952
	Cz	1.158	1.505	1.012	0.770	0.476	−2.711	5.027
	C4	−0.123	1.553	−0.065	−0.079	0.940	−4.114	3.868
	CP3	0.024	0.991	0.020	0.025	0.981	−2.522	2.571
	CPz	−0.224	0.499	−0.220	−0.449	0.672	−1.508	1.059
	CP4	0.253	1.728	0.122	0.146	0.889	−4.188	4.693
	P3	0.557	0.678	0.787	0.821	0.449	−1.186	2.299
	Pz	−0.306	0.345	−0.445	−0.886	0.416	−1.193	0.582
	P4	1.148	1.238	0.831	0.927	0.396	−2.035	4.331

*Note:* Dependent variable: MAE. Significance of * indicates *p* values when *p < 0.05.

## 4 Discussion

This study investigated the neural mechanisms underlying proprioceptive learning and cross‐transfer, focusing on changes of behavior with a custom‐made wrist joint evaluation device and EEGs (ERPs, MRCPs and ERSPs) following VMT. This study explored the phenomenon of contralateral transfer of training effects and the differences in cortical activation resulting from both VMT and CT. The behavioral study revealed that the visuomotor training involved rapid, precise, small‐amplitude flexion and extension movements of the wrist joint with visual guidance, which helped improve position sense. These findings are consistent with our previous research [24]. Unlike previous methods, this study utilized a stereoscopic maze for training, which is likely easier to implement in practical settings compared to robot‐assisted training. The findings demonstrate that VMT induces significant cortical adaptations in the central and parietal regions, facilitating proprioceptive improvements and interlimb transfer. In contrast, CT did not elicit similar neurophysiological changes, suggesting that VMT engages distinct neural mechanisms involving sensorimotor integration and interhemispheric communication.

### 4.1 Bilateral Brain Excitability May Be Induced by Visuomotor Training

This study analyzed brain activity in the central and parietal regions, and further investigated the cross‐transfer effect from the trained (right) wrist to the untrained (left) wrist through VMT. The EEG topography of the VMT group revealed that, during active right wrist movements, EEG activity in the parietal region was significantly decreased post‐training, leading to a pronounced reduction in the ERPs amplitudes. As VMT facilitated task familiarity and efficiency, less effort and cognitive resources were required, resulting in reduced parietal activation and decreased cortical excitability. The EEG topography for both the right and left wrists was consistent with the observed behavioral improvements, suggesting that the training modulated central excitability.

#### 4.1.1 MRCPs and Motor Preparation Efficiency

MRCP findings further support the notion that VMT optimizes proprioceptive‐motor processing. Before training, MRCP latency was shorter, and amplitude was higher, suggesting a higher cortical demand for movement execution. However, after VMT, a significant prolongation of MRCP latency was observed in C3 and C4 for the right wrist, while the amplitude at C3 and Cz significantly decreased. Similarly, MRCP latency significantly increased at C3, and amplitude decreased at Cz for the left wrist.

These findings suggest that VMT enhances movement efficiency by reducing the cortical resources required for motor execution. The increased MRCP latency may reflect a more refined motor preparation process, while the decreased amplitude indicates a shift toward more automatic and efficient motor execution. This is consistent with previous studies showing that reduced MRCP amplitude after training reflects improved motor efficiency and reduced cognitive effort in movement planning [31, 39].

Furthermore, the involvement of both C3 and C4 in the latency increase suggests that VMT enhances interhemispheric motor coordination, potentially facilitating cross‐transfer effects. The Cz amplitude reduction further implies a redistribution of cortical activation, reinforcing the idea that VMT strengthens sensorimotor integration mechanisms across both hemispheres.

#### 4.1.2 ERPs and Proprioceptive Processing

The changes in ERPs suggest that cross‐transfer may be induced by VMT. El‐Gohary et al. investigated the possible cross effects of visuomotor training on proprioceptive repositioning accuracy of the knee joint and demonstrated significant cross‐training effects on proprioceptive repositioning accuracy among healthy subjects [40]. In our study, the observed ERP amplitude reductions following VMT indicate enhanced neural efficiency in processing proprioceptive input. Specifically, right wrist testing resulted in a bilateral decrease in ERPs amplitudes in contralateral C3, CP3, and P3, as well as ipsilateral P4, while left wrist testing led to ERP reductions in C4, P4, and ipsilateral C3 and CP3. These results align with prior research showing that ERP modulation reflects cortical plasticity associated with sensory integration and cross‐limb transfer [40].

Regression analyses results suggest that sensorimotor cortical areas, particularly CP3, CP4, C3, and C4, play a crucial role in proprioceptive adaptation and interlimb transfer. The involvement of both contralateral and ipsilateral sensorimotor regions supports the notion that proprioceptive learning relies on dynamic interhemispheric interactions, reinforcing the idea that proprioceptive representations are not strictly lateralized but instead distributed across cortical networks. In contrast, no significant ERP‐behavior correlations were observed in the CT group, indicating that conventional training did not engage the same cortical mechanisms as VMT. Unlike VMT, which integrates visual feedback and task‐specific proprioceptive recalibration, conventional training appears to rely more on peripheral mechanisms without inducing cortical‐level adaptations.

The improvement in proprioceptive accuracy following VMT cannot be solely attributed to task repetition, as the participants underwent a familiarization phase before baseline testing. Additionally, the observed ERP‐behavior correlations suggest that the changes were due to genuine cortical adaptations rather than simple learning effects.

#### 4.1.3 ERSP and Sensorimotor Excitability

Central excitability refers to the ability of the central nervous system (CNS) to regulate sensorimotor responses, which is reflected in cortical oscillations [41]. Spectral analyses of EEG oscillations provide additional insights into the cortical mechanisms of proprioceptive adaptation. In our study, changes in α and β oscillations provided insights into the modulation of central excitability following VMT.

α‐ERS has been linked to better sensory integration and attentional modulation, particularly when proprioceptive tasks are performed in the absence of visual input [42]. In our study, α‐ERS in the PPC and S1 suggests that VMT enhances sensory gating, reducing interference from irrelevant sensory inputs and improving proprioceptive precision.

Additionally, the significant β‐ERD observed in the contralateral PMd indicates that VMT strengthens interhemispheric sensorimotor communication, facilitating cross‐limb proprioceptive transfer. Given the well‐established role of β‐ERD in sensorimotor adaptation and movement planning, its modulation in both the trained and untrained hemispheres suggests that proprioceptive learning extends beyond local cortical changes to involve widespread network reorganization [43]. The absence of significant β‐ERD modulation in the CT group further supports the idea that VMT, rather than repeated movement, plays a crucial role in modulating central excitability.

### 4.2 Conventional Training May Potentially Induce Fatigue

Fatigue is generally classified into central fatigue, originating from the central nervous system (CNS), and peripheral fatigue, arising from muscular inefficiency [44]. Central fatigue can be assessed via EEGs, typically manifested as increased cortical activation in the sensorimotor area [45] while peripheral fatigue is typically evaluated via surface electromyography (sEMG) [46, 47], reflecting neuromuscular inefficiency. In this study, behavioral analysis indicated that the CT group exhibited a decline in right wrist movement accuracy, suggesting fatigue‐related performance deterioration. However, EEG analysis did not reveal increased cortical activation—a hallmark of central fatigue—implying that the observed decline was likely due to peripheral fatigue.

#### 4.2.1 Central Fatigue in CT

The CT group demonstrated a decline in movement accuracy for the trained wrist, without significant improvements in the untrained wrist. Central fatigue is typically characterized by increased cortical activation in the sensorimotor region due to compensatory mechanisms attempting to maintain task performance [48].

EEG spectral analysis further supports this interpretation. α‐ERD at FC3 and CP3 was observed in the CT group, indicating diminished sensory gating and attentional modulation, both of which are associated with central fatigue [48]. Studies have shown that α‐ERD is linked to impaired sensory integration and attentional control during fatigued states [49]. Additionally, no significant β‐ERD changes were observed, suggesting that sensorimotor networks were not effectively engaged, reinforcing the likelihood that the observed fatigue was primarily peripheral.

#### 4.2.2 VMT Mitigates Central Fatigue

In contrast, VMT improved movement accuracy in both trained and untrained wrists, demonstrating effective interlimb proprioceptive transfer. Unlike the CT group, the VMT group exhibited α‐ERS at CP3 and CP4, suggesting enhanced sensory gating and attention regulation rather than fatigue‐related cognitive decline [50].

Furthermore, significant β‐ERD at contralateral S1 and PMd indicated improved sensorimotor integration and movement efficiency, reflecting neuroplasticity rather than fatigue‐induced decline [50, 51]. The presence of β‐ERD instead of β‐ERS suggests that the cortical adaptations induced by visuomotor training were more likely due to neuroplasticity rather than fatigue‐induced inefficiency [43]. The real‐time visual feedback in VMT may have played a key role in sustaining central excitability, preventing fatigue‐induced declines in movement accuracy [50].

#### 4.2.3 MRCP and ERP Evidence for Fatigue in CT Versus VMT

MRCPs are sensitive indicators of movement preparation and execution efficiency, making them useful for identifying central fatigue [31]. In the CT group, prolonged MRCP latency and reduced amplitude suggested fatigue‐induced declines in motor efficiency [39]. In contrast, the VMT group also exhibited prolonged MRCP latency, but this was accompanied by improved behavioral accuracy, indicating optimized motor planning efficiency rather than central fatigue [31].

Similarly, ERP amplitude reductions were observed in both CT and VMT groups, but their implications differ. In the CT group, ERP amplitude reductions likely reflect fatigue‐induced declines in sensory processing efficiency. Conversely, in the VMT group, they were associated with behavioral improvements, suggesting that training optimized sensory processing rather than indicated neural exhaustion [40].

### 4.3 Limitations

Several limitations should be acknowledged. First, this study focused on healthy participants, which limits the direct applicability of the findings to clinical populations. Future studies should explore whether similar neural adaptations occur in patients with neurological disorders. Second, the role of peripheral factors such as muscle fatigue was not fully assessed, warranting further investigation through EMG‐based analyses. Future research should extend these findings to clinical populations, such as stroke patients or individuals with neurodegenerative diseases, to evaluate the potential of VMT in facilitating proprioceptive recovery. Incorporating EMG assessments would help differentiate between central and peripheral factors influencing proprioceptive adaptation, while longitudinal studies could explore the long‐term effects of VMT, assessing whether repeated training leads to sustained proprioceptive and motor improvements over time.

## 5 Conclusions

Visuomotor training on the right wrist led to proprioceptive improvements in both the trained and untrained wrists, demonstrating a cross‐transfer effect. These findings suggest that visuomotor training could be used in rehabilitation protocols to improve proprioception in patients with neurological diseases, offering a less painful and more efficient method to restore motor control.

## Author Contributions

L.H. and Y.W. research project conception and design. Z.W. and Y.W. acquisition of data, Y.W. organization of data, Y.W. and T.L. analysis and interpretation of data. Y.W. and T.L. manuscript preparation.

## Conflicts of Interest

The authors declare no conflicts of interest.

## Supporting information


Data S1:

## Data Availability

The data underlying this article will be shared on reasonable request by the corresponding author.

## Appendix A Sample Size Estimation

Based on preliminary experiments, the Movement Accuracy Error (MAE) improved from approximately 2°–1.5°. The standard deviation (SD) was estimated to be one‐third of the MAE values, resulting in SDs of 0.66 and 0.5, respectively. Using these values, the effect size (Cohen's *d*) was calculated to be 0.838.

The following parameters were entered into G*Power for the calculation:

- Test family: *t*‐test.
- Statistical test: means: difference between two dependent means (matched pairs).
- Type of power analysis: a priori: compute required sample size—given w, power, and effect size.
- Effect size (*d*): 0.838.
- *α* (significance level): 0.05.
- Power (1‐β): 0.8.

The calculation yielded a required sample size of 14 participants per group. To account for potential attrition or variability, we recruited 15 subjects for each group in this study.

## Appendix B Custom‐Made Wrist Proprioception Evaluation Device Reliability and Validity

Custom‐made wrist proprioceptive evaluation device: In this study, a custom‐made wrist evaluation device was used to assess the proprioception function (the evaluation index was the movement accuracy error, MAE). This device is equipped with a built‐in magneto sensitive angle sensor, and the movement angle can be displayed on an external monitor with a precision of 0.01°. The subject was seated on the left side of the device with the right forearm placed naturally on the forearm tray on the workbench. Adjust the seat to the appropriate height, so that the angle between the torso and the upper arm was about 60°, and the angle between the forearm and the upper arm was about 90°. Hold the handle with fingers, and the starting position was that the hand and forearm point straight ahead. The subject's wrist can perform passive flexion movement (pushed by the experimenter) in the horizontal plane through the workbench, and the subject can also perform active flexion movement. The angle at which the elbow joint moved from the starting position (neutral position) to the end of the movement (either active or passive) can be shown via an external display. The reliability and validity of the test equipment have been verified before the formal start of the experiment.

1. Custom‐made device reliability test.

The intra—class correlation coefficient (ICC) test was conducted on the self—made proprioceptive sensation evaluation device for the wrist joint to assess the test—retest reliability. A total of four subjects were selected. For each subject, an arbitrary angle between 10° and 15° was chosen, and the wrist joint was moved five times to the left and five times to the right respectively. Then, the subjects were asked to match the perceived angles with their wrist joints.

After the test, the intra—class correlation coefficient for the right wrist joint flexion (to the left) was 0.884, with a 95% confidence interval of 0.607–0.991; the intra—class correlation coefficient for the left wrist joint flexion (to the right) was 0.898, with a 95% confidence interval of 0.660–0.992; the overall intra—class correlation coefficient of the device was 0.882, with a 95% confidence interval of 0.723–0.971. Therefore, it is concluded that the custom‐made proprioceptive wrist proprioceptive evaluation device has high reliability (Table A1).

**TABLE A1:** Reliability test of the custom‐made wrist proprioceptive evaluation device.

Angle number	Direction	ICC	ICC 95% CI	*p*
4	Left	0.884	0.607–0.991	< 0.001
4	Right	0.898	0.660–0.992	< 0.001
8	Left and right	0.882	0.723–0.971	< 0.001

2 Custom‐made device validity test.

The validity of the Custom‐made wrist proprioceptive evaluation device was tested by comparing the perforation degree of the device with the electronic display. Select the odd punching degree of the device to the left (3°, 5°, 7°, 9°, 11°, 13°, 15°, and 17°), move the lever of the device to the left, compare the punching degree with the electronic display degree, and conduct correlation analysis; Select the even punching degree of the device to the right (2°, 4°, 6°, 8°, 10°, 12°, 14°, and 16°), move the lever of the device to the right, compare the punching degree with the electronic display degree, and conduct correlation analysis. After correlation analysis, the left, right and comprehensive correlation of the equipment were highly correlated, so it was concluded that the self‐made proprioceptive evaluation device for the wrist joint had high validity (Table A2).

**TABLE A2:** Validity test of the custom‐made wrist proprioceptive evaluation device.

Angle number	Direction	Pearson	*p*
8	Left	0.999	< 0.001
8	Right	0.999	< 0.001
16	Left and right	0.999	< 0.001
