# Supplementary material for: Visuomotor Training to Enhance Proprioception of Contralateral Wrist Based on the Cross‐Transfer Effect
Source: CNS Neurosci Ther. 2025 Sep 10;31(9):e70504. doi: 10.1111/cns.70504 (PMC12423547; doi:10.1111/cns.70504)
Supplement: Supplementary file 1 — Data S1: [file CNS-31-e70504-s001.docx]

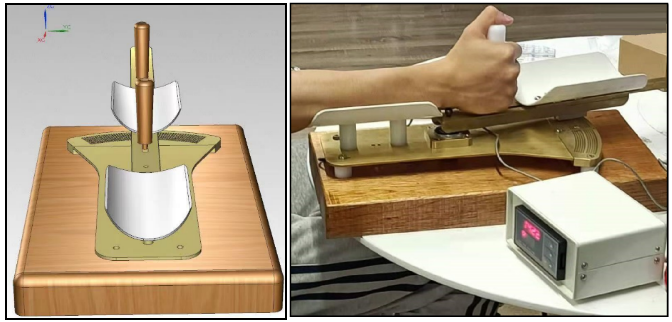


Fig. 1. Custom-made wrist proprioception evaluation device


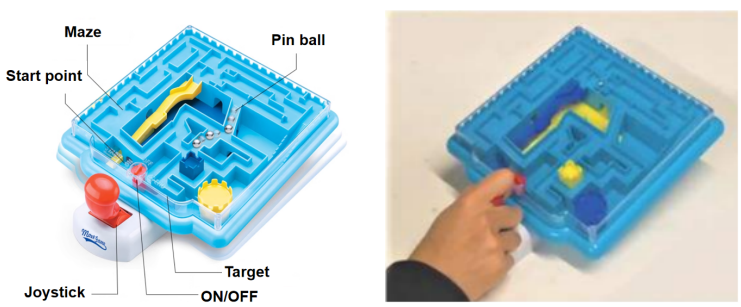


Fig. 2. Wrist-manipulated 3D maze. A wrist-operated joystick can tilt the panel at different angels, allowing the participant to guide the pin ball through the maze.

1. Experimental design


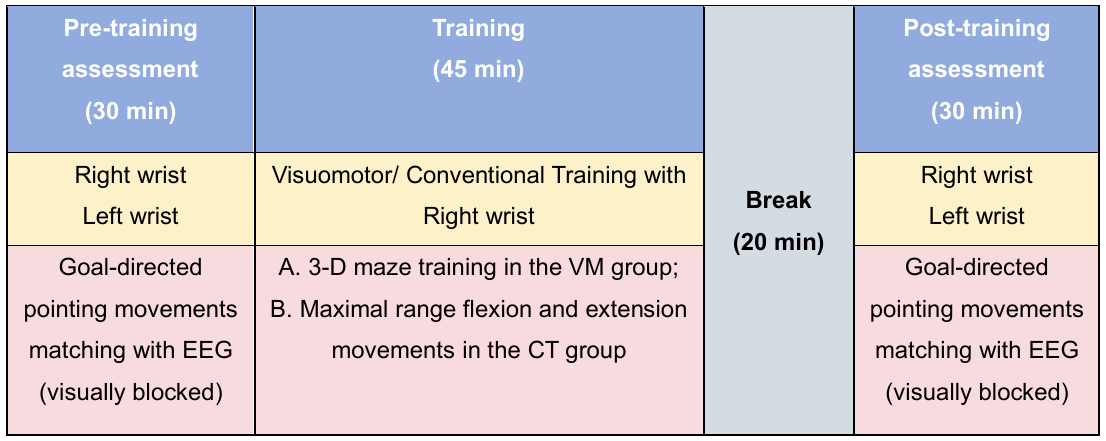


- 1. Pre-/post-training behavioral tests


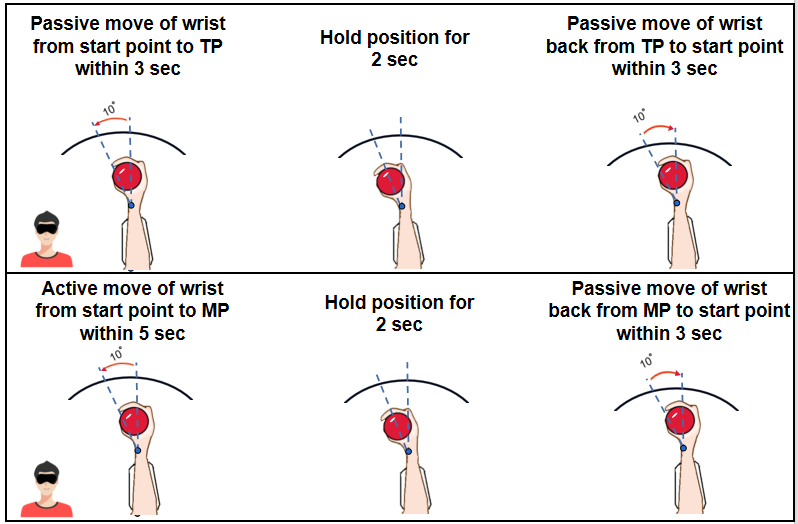


Fig. 3. Schemata of experimental design (Fig.3 A) and the procedure of pre-/post-training behavioral tests (Fig.3 B). The participants were visually blocked during behavioral tests.


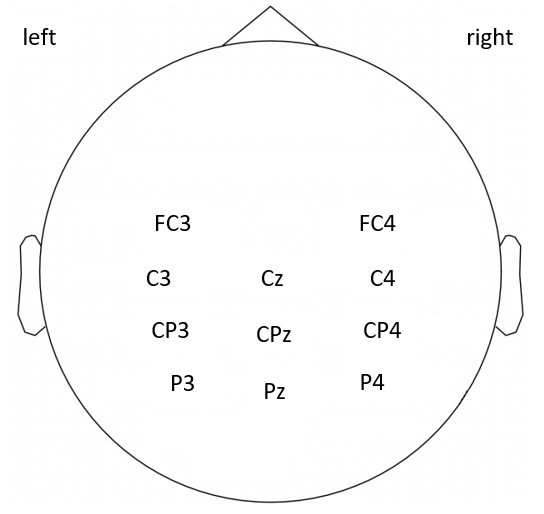


Fig. 4. Placement of electrodes. EEGs were recorded at FC3, FC4, C3, Cz, C4, CP3, CPz, CP4, P3, Pz and P4.


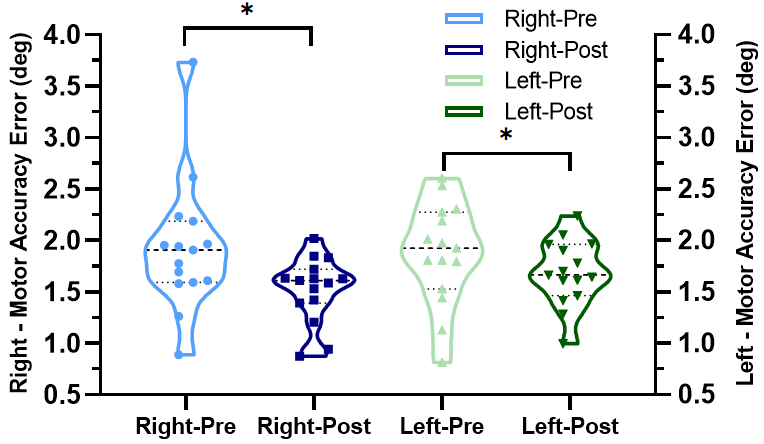


Fig. 5. Violin plot indicating the distribution of the MAE at pre-/post-training for the right/left wrist of the VM group. Dot lines represent the 25^th^ and 75^th^ percentiles. Dash line represents the median. Each point in the violin plot represents one participant. * *p* < 0.05


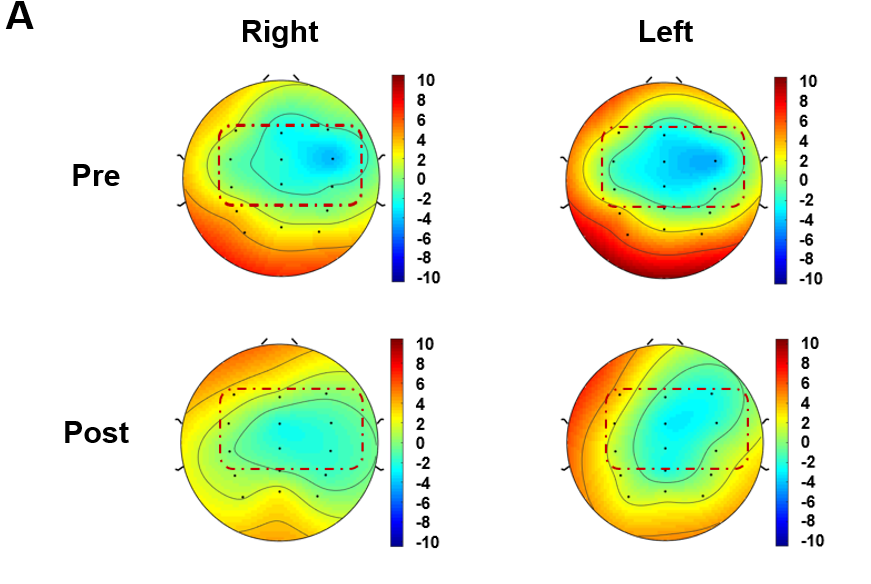


Fig. 6 A. Topographical 2D scalp maps at pre-/post-training for the left/right wrist of the VM group. Red box: sensorimotor area of the parietal region.


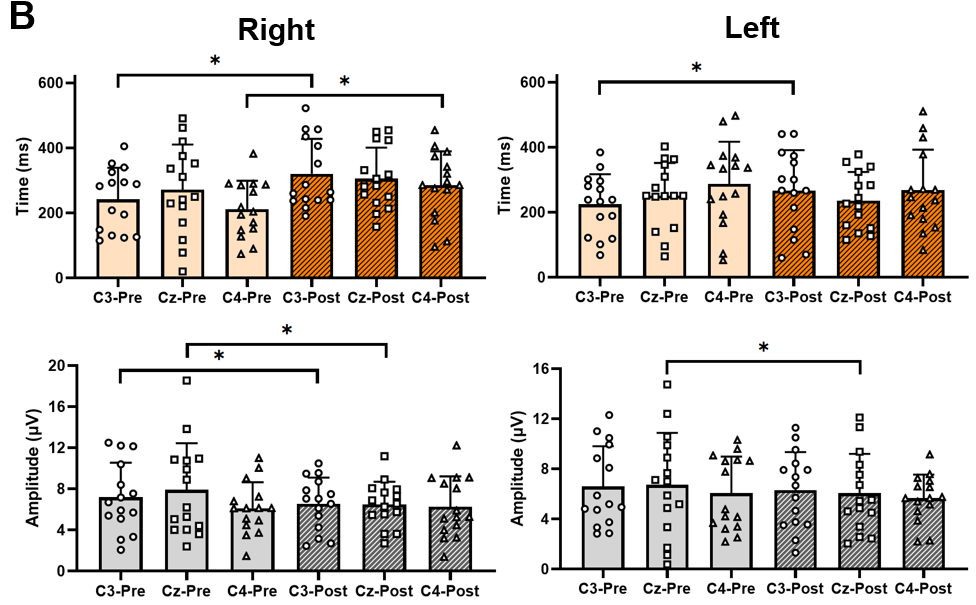


Fig. 6 B. MRCPs latency and amplitude at C3, Cz and C4 pre-/post-training for the right/left wrist of the VM group. Each point in the bar chart represents one participant. * *p* < 0.05


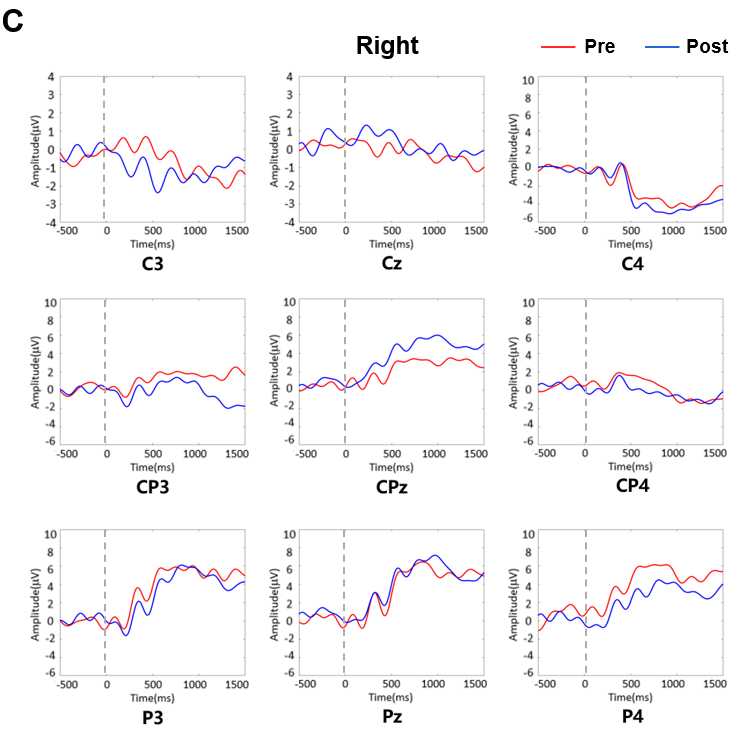


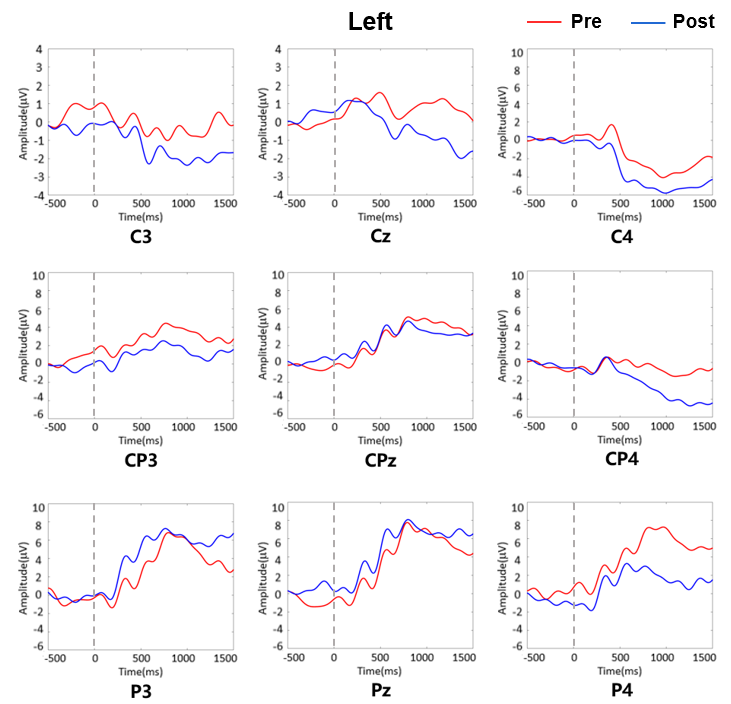


Fig. 6 C. ERPs (C3, Cz, C4, CP3, CPz, CP4, P3, Pz, P4) at pre-/post-training for the right/left wrist of the VM group. Red line: pre-training. Blue line: post-training. Dash line: trigger.


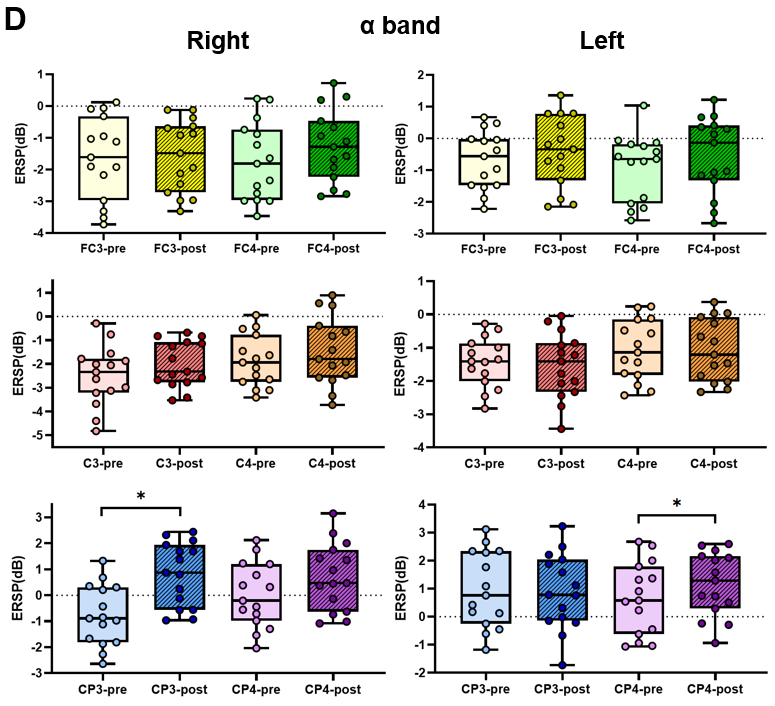


Fig. 6 D. α-frequency ERSP (FC3, FC4, C3, C4, CP3, CP4) at pre-/post-training for the right/left wrist of the VM group. Each point in the bar chart represents one participant. * *p* < 0.05


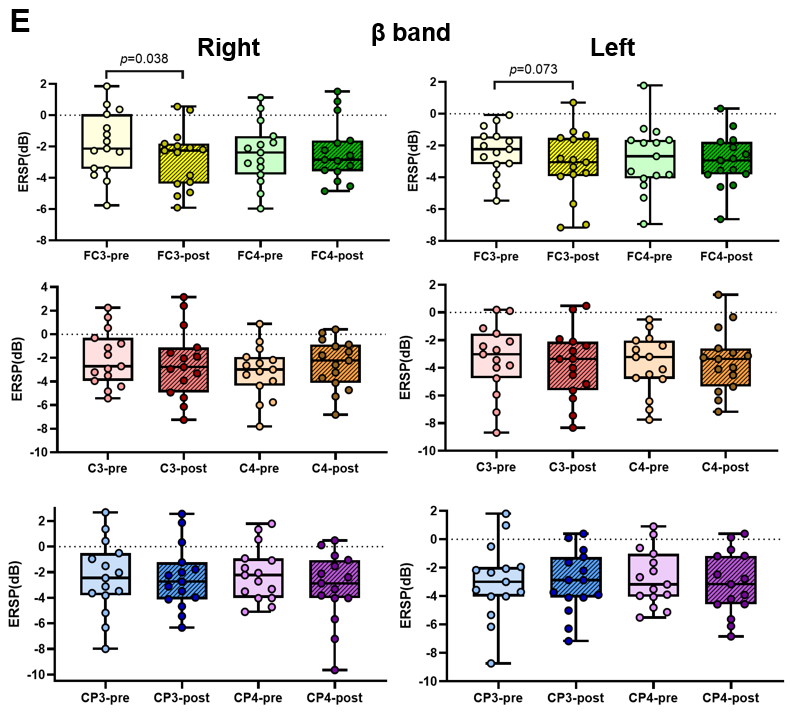


Fig. 6 E. β-frequency ERSP (FC3, FC4, C3, C4, CP3, CP4) at pre-/post-training for the right/left wrist of the VM group. Each point in the bar chart represents one participant. * *p* < 0.05


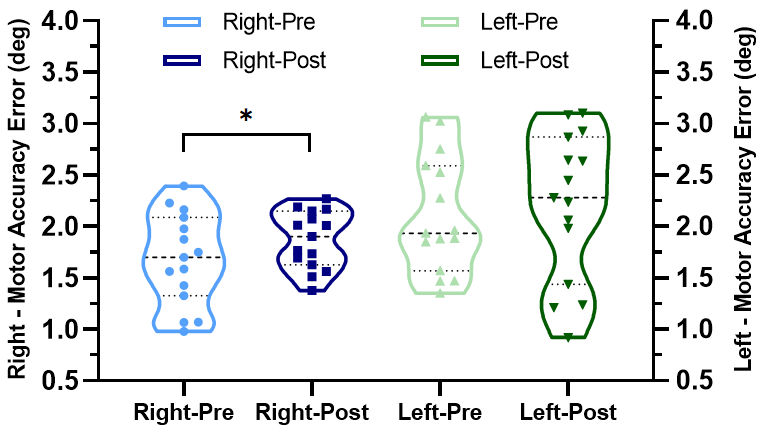


Fig. 7. Violin plot indicating the distribution of the MAE at pre-/post-training for the right/left wrist of the CT group. Dot lines represent the 25^th^ and 75^th^ percentiles. Dash line represents the median. Each point in the violin plot represents one participant. * *p* < 0.05


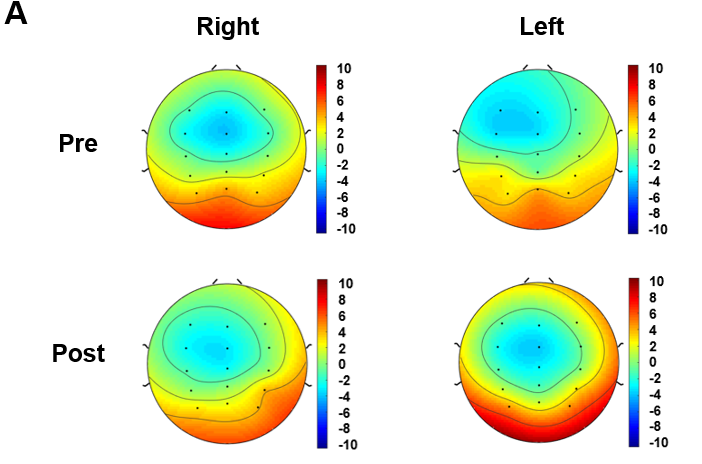


Fig. 8 A. Topographical 2D scalp maps at pre-/post-training for the left/right wrist of the VM group.


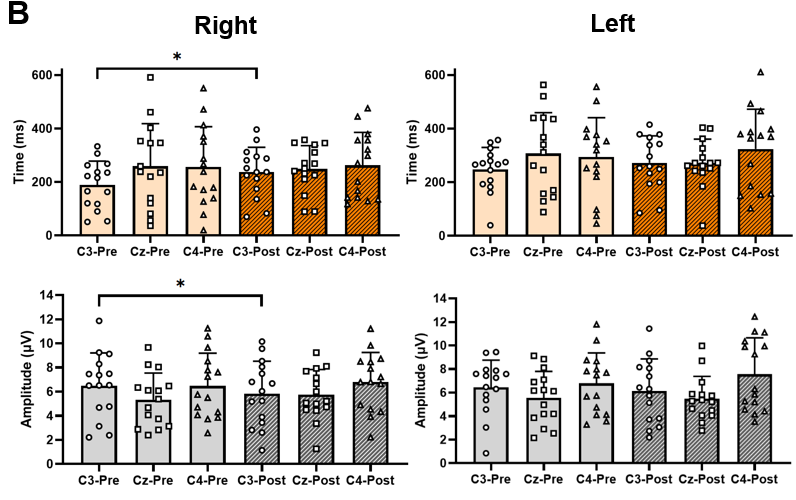


Fig. 8 B. MRCPs latency and amplitude at C3, Cz and C4 pre-/post-training for the right/left wrist of the CT group. Each point in the bar chart represents one participant. * *p* < 0.05


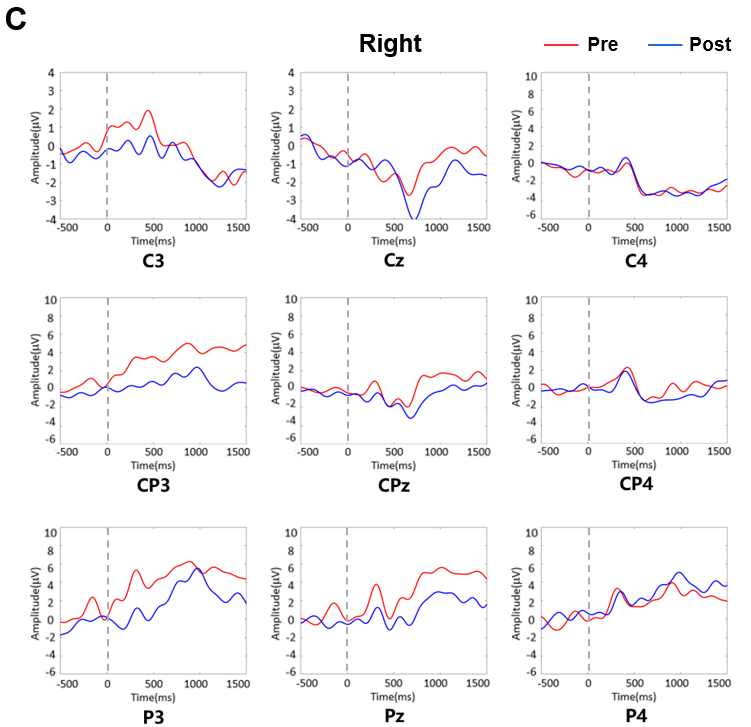


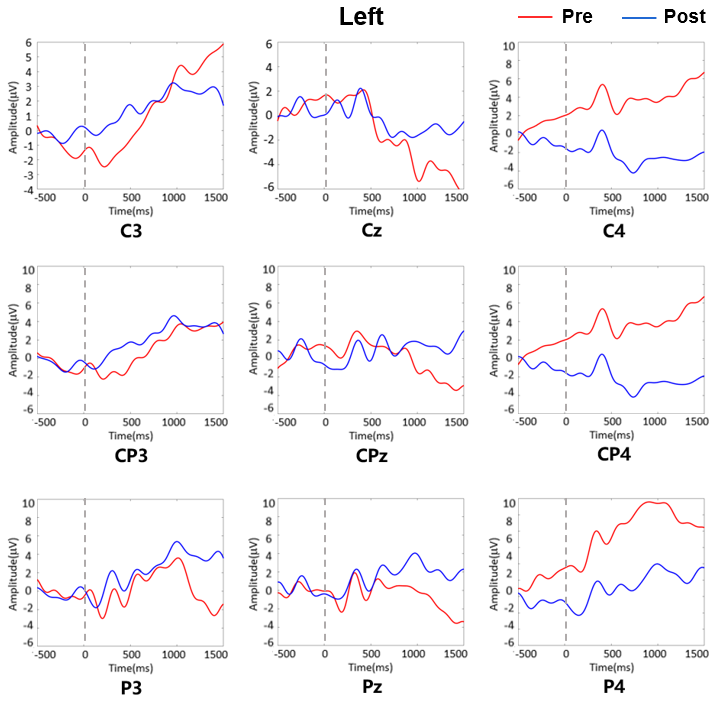


Fig. 8 C. ERPs (C3, Cz, C4, CP3, CPz, CP4, P3, Pz, P4) at pre-/post-training for the right/left wrist of the CT group. Red line: pre-training. Blue line: post-training. Dash line: trigger.


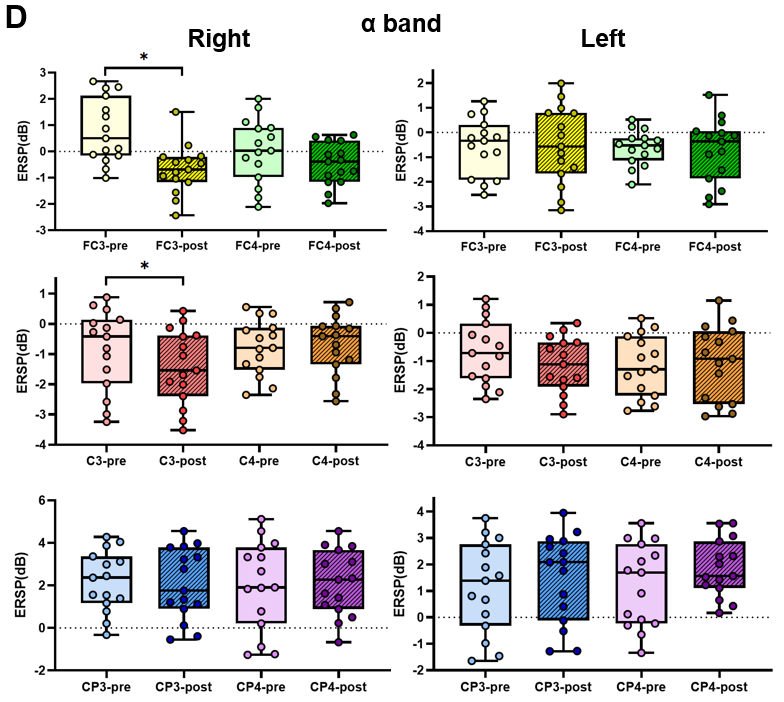


Fig. 8 D. α-frequency ERSP (FC3, FC4, C3, C4, CP3, CP4) at pre-/post-training for the right/left wrist of the CT group. Each point in the bar chart represents one participant. * *p* < 0.05


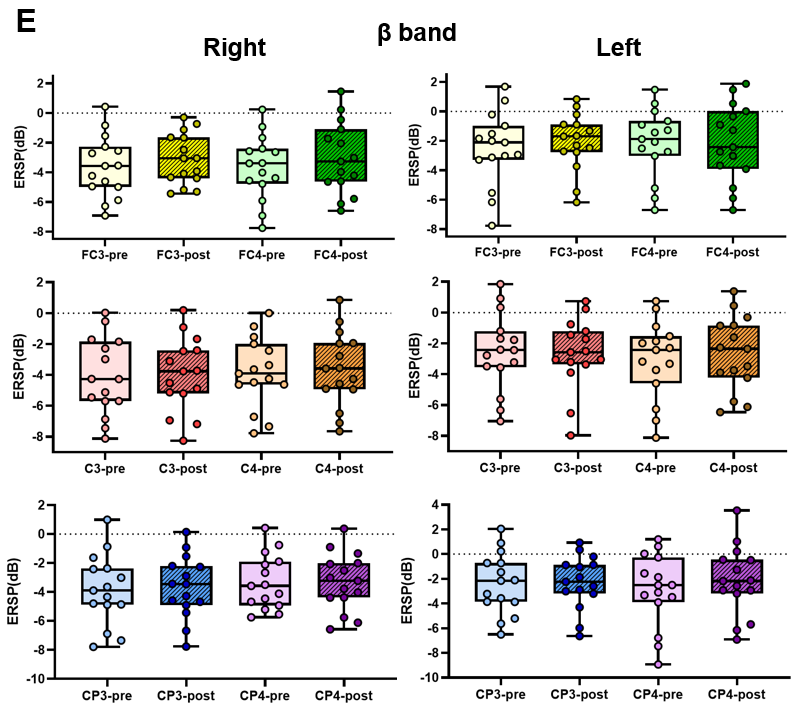


Fig. 8 E. β-frequency ERSP (FC3, FC4, C3, C4, CP3, CP4) at pre-/post-training for the right/left wrist of the CT group. Each point in the bar chart represents one participant. * *p* < 0.05


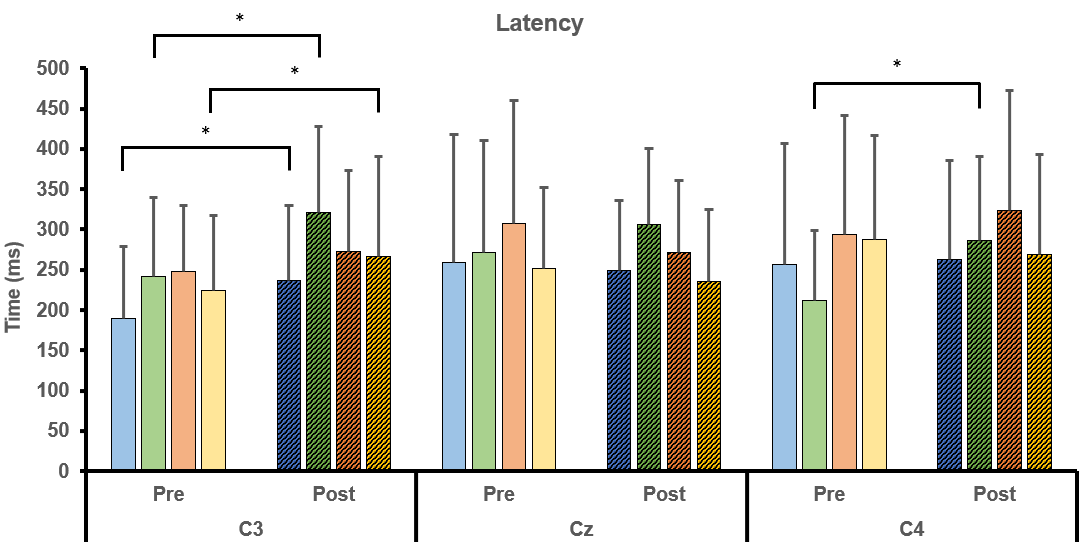


Fig. 9. Intra-/inter-group comparison for VM and CT group on latency.


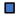
CT, right wrist.
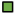
VM, right wrist.
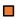
CT, left wrist.
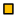
VM, left wrist. **p* < 0.05


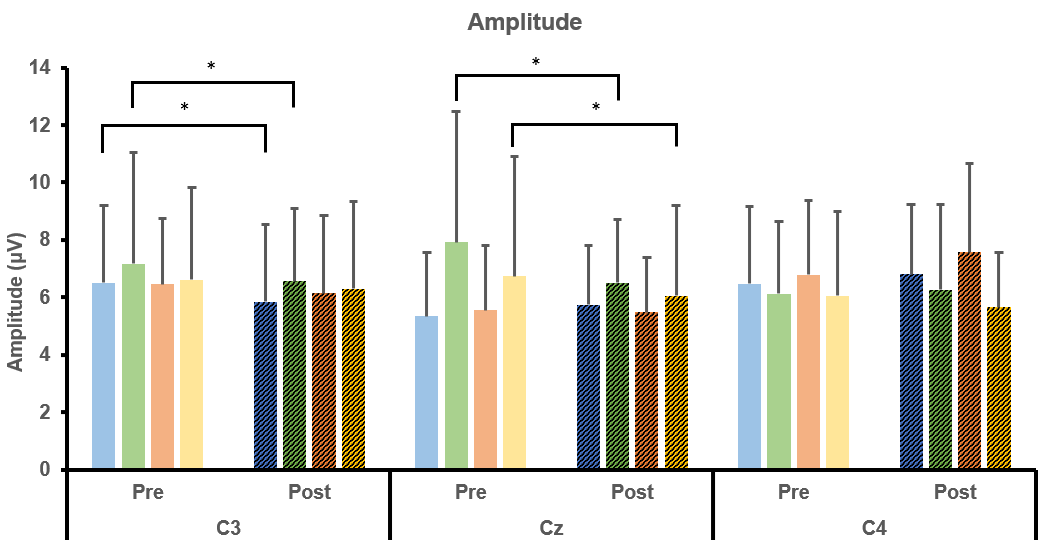


Fig. 10. Intra-/inter-group comparison for VM and CT group on amplitude.


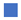
CT, right wrist.
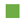
VM, right wrist.
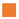
CT, left wrist.
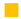
VM, left wrist. **p* < 0.05
